# The global, regional, and national burden of type 2 diabetes mellitus attributable to low physical activity from 1990 to 2021: a systematic analysis of the global burden of disease study 2021

**DOI:** 10.1186/s12966-025-01709-8

**Published:** 2025-01-16

**Authors:** Jiehua Wei, Luying Fan, Zixuan He, Senmao Zhang, Ying Zhang, Xidi Zhu, Fan Xia, Xinli Song, Lizhang Chen, Zhiyong Zou, Tingting Wang

**Affiliations:** 1 Department of Epidemiology and Health Statistics, Xiangya School of Public Health, Central South University, 110 Xiangya Road, Changsha, 410078 Hunan China; 2 College of Public Health, Chongqing Medical University, Chongqing, 400016 China; 3 School of public health, Harbin Medical University, Harbin, 150081 China; 4 Stroke Biological Recovery Laboratory, Spaulding Rehabilitation Hospital, the teaching affiliate of Harvard Medical School, Boston, USA; 5 Hunan Provincial Key Laboratory of Clinical Epidemiology, Xiangya School of Public Health, Central South University, Changsha, 410078 Hunan China; 6 Institute of Child and Adolescent Health and National Health Commission Key Laboratory of Reproductive Health, Peking University, Beijing, China

**Keywords:** Disability-adjusted life years, Global burden of disease, Mortality, Physical activity, Type 2 diabetes mellitus

## Abstract

**Background:**

Low physical activity (LPA) is a leading risk factor for type 2 diabetes mellitus (T2DM). We examine the temporal and spatial trends in the burden of T2DM attributable to LPA at the global, regional, and country scales.

**Methods:**

Data were obtained from the Global Burden of Disease Study 2021. The numbers of deaths and disability-adjusted life years (DALYs) of LPA-related T2DM, and the corresponding age-standardized mortality rate (ASMR) and age-standardized DALYs rate (ASDR) were compared across regions and countries by age, sex, and sociodemographic index (SDI). The annual percentage changes (EAPCs) in the ASMR or ASDR were calculated to quantify temporal trends from 1990 to 2021. We also quantified the relationship between SDI and the ASMR and ASDR of T2DM attributable to LPA.

**Results:**

Globally, the number of T2DM deaths and DALYs attributable to LPA were approximately 0.15 million and 5.52 million respectively in 2021, which more than doubled compared to 1990. Over the past 32 years, the global EAPCs of ASMR and ASDR were 0.26 (95% *CI*: 0.20, 0.31) and 0.97 (95% *CI*: 0.93, 1.02), respectively. The ASMR or ASDR had a reverse U-shaped relationship with the SDI, with the most severe burden observed in the low-middle and middle SDI regions. The age group older than 60 years had the highest rate of DALYs for LPA-related T2DM in 2021, while the 25–44 age group showed the largest increase between 1990 and 2021.

**Conclusions:**

Over the past 32 years, the global burden of LPA-related T2DM has continued to increase at an alarming rate in almost all countries, particularly in regions with low-middle and middle SDI. Substantial increases in national action are urgently needed to target elder populations especially in low-middle and middle SDI regions, and special efforts should be made to promote physical activity in young adults with LPA.

**Supplementary Information:**

The online version contains supplementary material available at 10.1186/s12966-025-01709-8.

## Introduction

Type 2 diabetes mellitus (T2DM) stands as among the swiftly burgeoning chronic non-communicable ailments on a global scale. In 2021, an estimated 529 million individuals worldwide were afflicted with T2DM, with projections suggesting that this figure will soar to 1.31 billion by the year 2050 [1]. Not only is the number of new cases of T2DM increasing rapidly after age 55 years, but the disease is now increasing in younger age groups (aged ≤ 40 years), resulting in a greater burden of disease at earlier ages [2]. Healthcare expenditures related to T2DM were estimated at $966 billion globally in 2021, with forecasts indicating a rise to over $1,054 billion by 2045 [3]. Thus, efforts to mitigate the enormous social, health, and economic burdens of T2DM have become a primary public health priority.

Previous studies have indicated that physical activity is regarded as fundamental for both the prevention and management of T2DM [4]. Considerable prospective evidence for T2DM prevention has shown that moderate-to-vigorous physical activity is beneficial for controlling glycemia and substantially decreasing cardiovascular and overall mortality risk [5, 6]. In contrast, low physical activity (LPA, not meeting specified physical activity guidelines) could be the major factor associated with an increased risk of developing T2DM [7]. In 2012, a study reported that LPA was directly responsible for 7% of the T2DM burden globally, and the LPA-related T2DM burden has showed an aggravating trend due to increasing levels of physical inactivity worldwide [8]. In addition, annual LPA-related deaths and healthcare costs exceed 5 million and $67.5 billion, respectively [9, 10]. Thus, physical inactivity emerges as a significant public health concern, with the WHO ranking it as the fourth leading risk factor for mortality over the past decade [11].

Given the upwards trends in T2DM and LPA prevalence globally, understanding the detailed disease burden of T2DM attributable to LPA is highly importance, and will guide sport resource allocation and provide guidance for T2DM prevention. However, to date, the burden of T2DM attributable to LPA and its global and regional spatiotemporal trends have not been comprehensively estimated. Only one article reported the global disease burden attributable to LPA based on GBD2019 [12], but it did not systematically assess the global, regional, or country burden estimates of LPA-related T2DM. Furthermore, the existing literature lacks a thorough investigation into the correlation between LPA-related T2DM and SDI. The Global Burden of Diseases Study (GBD) 2021 is a systematic and comprehensive global epidemiological study [13, 14]. It quantifies the health burden attributable to 371 diseases and integrates 88 associated risk factors on a global scale. This study utilized the GBD 2021 dataset to analyze the spatiotemporal trends in the burden of T2DM attributed to LPA from 1990 to 2021 across different age groups, genders, regions, and SDI. Furthermore, we examined the associations between deaths and disability-adjusted life years (DALYs) related to LPA-related T2DM with the SDI. These findings may inform the development of contemporary public health policies targeting the reduction of the burden associated with LPA-related T2DM.

## Materials and methods

### Data collection

The data concerning the global burden of T2DM attributed to LPA for this study were sourced from the GBD 2021 study [13, 14]. Data on annual deaths related to T2DM, age-standardized mortality rates (ASMR), DALYs, and age-standardized disability-adjusted life year rates (ASDRs) attributable to LPA in 204 countries and territories from 1990 to 2021 were extracted. ASMR is a measure that allows for comparisons of mortality rates across populations with different age structures by adjusting for age differences. ASDR is similar to ASMR but accounts for both mortality and disability, adjusted for age structure. This extraction encompassed both sexes and various age groups, represented in successive 5-year intervals from 25 to 29 to 90–94. The data retrieval was conducted from the online Global Health Data Exchange website (http://ghdx.healthdata.org/gbd-results-tool). In GBD 2021, data on the global burden of T2DM were estimated from a large and growing number of data sources, including household survey data, epidemiological surveillance data, disease registry data, clinical informatics data, and other health-related data sources [15]. Based on the socio-demographic index (SDI), the 204 countries and territories were classified into five regions: high SDI (> 0.81), high-middle SDI (0.70–0.81), middle SDI (0.61–0.69), low-middle SDI (0.46–0.60), and low SDI (< 0.46). The SDI was computed using lag-distributed income per capita, total fertility rate among individuals under the age of 25, and mean education level for individuals aged 15 and older, which was a composite index to present a geographical location’s development status [16]. The SDI ranges from 0 to 1, and a greater SDI value indicates a higher socioeconomic level. In addition, these countries and territories were divided into 21 GBD regions according to socioeconomic similarities and geographical proximity. This study adhered to the Strengthening the Reporting of Observational Studies in Epidemiology (STROBE, Supplementary Material 1). This study protocol was exempted from ethical review by the Ethical Board of Xiangya School of Public Health, Central South University, because the GBD data were de-identified and presented in aggregate form.

### Definitions of T2DM and LPA

The GBD study defined T2DM as a fasting plasma glucose ≥ 126 mg/dL (7 mmol/L) or current treatment (insulin or anti-diabetic drugs), with the E11-E11.1 and E11.3-E11.9 codes in the International Classification of Diseases version 10.

The primary sources of data on LPA for the GBD study included the Global Physical Activity Questionnaire, the International Physical Activity Questionnaire, and various other survey instruments [16]. In the GBD study, physical activity lasting a minimum of ten minutes for adults aged 25 years and above is assessed across all life domains, including leisure/recreation, work/household, and transportation. The total metabolic equivalent-minutes per week are calculated based on the frequency, duration, and intensity of activity. The metabolic equivalent (MET) was defined as the ratio of the working metabolic rate to the resting metabolic rate. One MET was equivalent to the energy cost of sitting quietly, approximately 1 kcal/kg/hour, or to the oxygen cost of sitting quietly, approximately 3.5 ml/kg/min.

Based on the quartiles of the total global MET-minutes/week, physical activity levels were classified into four categories: inactive (< 600 MET-minutes per week), low active (600–3999 MET-minutes per week), moderately active (4000–7999 MET-minutes per week), and highly active (≥ 8000 MET-minutes per week) [15, 16]. In the GBD 2021 study, the theoretical minimum risk exposure level for physical inactivity was set between 3600 and 4400 MET-minutes per week. Consequently, LPA was defined as less than 3600 MET-minutes per week [16].

### Estimation of T2DM burden and attribution burden

The number of deaths, ASMR, DALYs, and ASDR were used to quantify the T2DM burden at the global, regional, and national levels. All estimates were reported with 95% uncertainty intervals (UIs). To ensure robustness and reliability, all calculations were conducted 500 times to generate draw-level estimates in GBD 2021. The final estimates were derived as the mean value across these 500 draws, and 95% UIs are represented by the 2.5th and 97.5th percentile values across the draws [13]. All the T2DM-specific estimates were sourced from the GBD 2021 study. This study applied complex statistical models, and the specific methods for estimating T2DM burden have been detailed described in the GBD 2021 Diseases and Injuries study’s supplementary appendix [15]. In brief, the estimates of cause-specific mortality and years of life lost were derived through standard cause of death ensemble modelling, utilizing vital registration and surveillance data. Likewise, the comprehensive methodology for assessing the burden of T2DM attributable to LPA was described upon in the GBD 2021 Risk Factors study’s supplementary appendix [16].

### Statistical analysis

To gauge the trend of the age-standardized rate (ASR) over the specified time frame from 1990 to 2021, the estimated annual percentage change (EAPC) was utilized. EAPC is a statistical measure used to quantify the average annual rate of change in a specific metric over time. It represents the percentage change in the metric per year. The EAPC was computed using the following linear regression model:

$$\:In\left(ASR\right)=\:\alpha\:+\beta\:x+\epsilon\:$$

$$\:EAPC=100\times\:(exp\left(\beta\:\right)-1)$$

where *x* is the calendar year, *ε* is the error term, and *β* describes the increasing or decreasing age-standardized rate trend. The logarithmic transformation of ASR allows for the modeling of relative changes in rates over time, ensuring that the trend follows a linear pattern. This approach is widely used in epidemiological studies to estimate percentage changes in rates over time [17, 18]. The EAPC and its 95% confidence intervals (*CIs*) can be obtained from this model. If both the EAPC and its 95% *CI* were > 0, the trend was considered to be increasing, a decreasing trend was considered to be < 0, and a stable trend was considered to be present if the 95% *CI* included 0. In addition, restricted cubic spline regression, a parametric approach that allows for modeling and quantifying potential non-linear relationships, was used to analyze the association between the EAPCs of T2DM burden attributable to LPA and the SDI in 2021 at the national level. We also applied locally estimated scatterplot smoothing (LOESS) regression to examine the shape of the association between T2DM burden attributable to LPA and the SDI across 21 regions from 1990 to 2021 [19]. Statistical significance was defined as a two-sided *P*-value less than 0.05. All statistical analyses were conducted using R program version 4.2.2.

## Results

### Global T2DM burden attributed to LPA and its temporal trends

Over the past 32 years, the global population grew by approximately 47.92% (7.89 billion in 2021). Globally, the number of T2DM deaths attributable to LPA was approximately 0.15 million (95% UI: 0.07, 0.23), with corresponding DALYs totaling around 5.52 million (95% *UI*: 2.41, 8.64) in 2021. The female-male ratio of T2DM deaths attributable to LPA was approximately 1.80, while the female-male ratio of DALYs attributable to LPA for T2DM was approximately 1.64. From 1990 to 2021, the number of T2DM deaths attributed to LPA more than doubled, and the burden of DALYs attributable to LPA more than tripled. Moreover, corresponding ASMR and ASDR also exhibited increases, with EAPCs of 0.26 (95% *CI*: 0.20, 0.31) and 0.97 (95% *CI*: 0.93, 1.02), respectively (Table 1).

**Table 1:** The global type 2 diabetes mellitus burden attributable to low physical activity in 1990 and 2021 and the temporal trends from 1990 to 2021

Characteristics	1990				2021				EAPC (1990–2021)	
	Deaths cases No. (95%UI)	ASMR per 10^5^ No. (95%UI)	DALYs No. (95%UI)	ASDR per 10^5^ No. (95%UI)	Deaths cases No. (95%UI)	ASMR per 10^5^ No. (95%UI)	DALYs No. (95%UI)	ASDR per 10^5^ No. (95%UI)	ASMR No. (95%CI)	ASDR No. (95%CI)
Global	55,801 (24049, 85577)	1.64 (0.71, 2.51)	1,755,082 (756859, 2694173)	46.06 (19.9, 70.69)	149,214 (65194, 228318)	1.8 (0.79, 2.75)	5,523,050 (2407128, 8638535)	64.27 (28.01, 100.49)	0.26 (0.2, 0.31)	0.97 (0.93, 1.02)
**Sex**										
Male	7497 (18297, 27922)	0.53 (1.28, 1.95)	264,577 (621637, 973518)	15.55 (36.48, 56.64)	23,392 (54105, 84094)	0.65 (1.5, 2.34)	907,065 (2094650, 3290688)	22.89 (52.92, 83.10)	0.48 (0.42, 0.54)	1.08 (1.03, 1.13)
Female	16,920 (37504, 57636)	0.85 (1.89, 2.91)	495,920 (1133445, 1751492)	23.65 (53.94, 83.42)	42,059 (95108, 145455)	0.90 (2.03, 3.11)	1,487,012 (3428400, 5330556)	32.27 (74.38, 115.78)	0.18 (0.13, 0.24)	0.95 (0.91, 0.99)
**SDI region**										
High SDI	14,763 (6325, 22565)	1.31 (0.56, 2.02)	431,738 (187455, 669806)	38.79 (16.84, 60.23)	21,403 (8958, 33305)	0.88 (0.37, 1.37)	1,057,953 (470182, 1697065)	52.1 (23.03, 84.44)	-1.64 (-1.84, -1.44)	0.74 (0.61, 0.86)
High-middle SDI	10,833 (4664, 16594)	1.28 (0.56, 1.95)	348,797 (151116, 533856)	36.66 (15.98, 56.34)	25,209 (11305, 38576)	1.3 (0.58, 1.98)	950,867 (416024, 1492242)	48.14 (21.04, 75.47)	0.05 (-0.03, 0.13)	0.79 (0.74, 0.85)
Middle SDI	16,217 (6985, 24854)	2.03 (0.87, 3.13)	548,693 (237748, 847190)	56.85 (24.6, 88.05)	55,677 (24646, 84455)	2.32 (1.03, 3.53)	2,007,029 (877454, 3117402)	75.81 (33.18, 117.39)	0.42 (0.36, 0.47)	0.82 (0.77, 0.86)
Low-middle SDI	10,383 (4421, 15795)	2.19 (0.93, 3.34)	318,972 (136875, 490980)	56.9 (24.41, 87.64)	37,852 (16402, 58173)	3.22 (1.39, 4.96)	1,204,356 (519769, 1871158)	88.59 (38.29, 137.73)	1.38 (1.31, 1.45)	1.46 (1.41, 1.5)
Low SDI	3512 (1430, 5471)	2.04 (0.83, 3.19)	103,907 (43162, 161519)	50.22 (20.71, 78.34)	8886 (3673, 14049)	2.33 (0.96, 3.70)	295,801 (125367, 470146)	62.74 (26.4, 99.23)	0.48 (0.37, 0.58)	0.68 (0.61, 0.74)
**GBD region**										
Andean Latin America	242 (99, 381)	1.33 (0.54, 2.09)	7167 (2982, 11530)	36.48 (15.18, 58.09)	930 (393, 1496)	1.65 (0.69, 2.64)	31,398 (13435, 50134)	53.89 (23.08, 86.04)	0.61 (0.48, 0.75)	1.18 (1.1, 1.27)
Australasia	324 (134, 493)	1.42 (0.59, 2.16)	9319 (4137, 14744)	39.59 (17.42, 62.96)	667 (288, 1045)	1.09 (0.46, 1.70)	25,141 (11117, 40710)	47.32 (20.97, 76.47)	-1.18 (-1.5, -0.87)	0.41 (0.32, 0.5)
Caribbean	1116 (478, 1709)	4.64 (1.99, 7.09)	32,440 (13845, 50273)	126.71 (54.09, 196.08)	1975 (853, 3120)	3.63 (1.57, 5.73)	72,902 (31759, 117916)	135.27 (58.91, 219.06)	-0.92 (-1, -0.83)	0.09 (0.03, 0.15)
Central Asia	222 (94, 348)	0.51 (0.22, 0.8)	8843 (3852, 14046)	19.30 (8.43, 30.67)	649 (275, 1037)	0.91 (0.39, 1.45)	29,893 (12835, 48144)	37.44 (16.05, 60.33)	1.71 (1.31, 2.1)	2.02 (1.77, 2.27)
Central Europe	1525 (658, 2382)	1.06 (0.46, 1.66)	57,430 (25224, 92466)	38.46 (16.85, 61.44)	3250 (1389, 4958)	1.34 (0.57, 2.04)	121,418 (53313, 195290)	53.07 (23.28, 85.99)	0.98 (0.8, 1.16)	1.18 (1.09, 1.27)
Central Latin America	2512 (995, 3863)	3.48 (1.39, 5.39)	78,188 (32329, 122249)	96.42 (39.05, 150.46)	7819 (3420, 12123)	3.28 (1.43, 5.09)	266,350 (114002, 425533)	106.24 (45.54, 169.23)	-0.4 (-0.71, -0.08)	0.08 (-0.23, 0.39)
Central Sub-Saharan Africa	575 (232, 940)	3.79 (1.55, 6.15)	15,981 (6280, 26085)	83.47 (33.29, 134.85)	1425 (592, 2408)	3.88 (1.64, 6.46)	45,016 (18542, 76016)	94.06 (40.1, 157.87)	-0.04 (-0.13, 0.06)	0.30 (0.20, 0.40)
East Asia	6174 (2527, 9659)	0.95 (0.40, 1.47)	255,761 (108188, 403667)	31.49 (13.27, 49.35)	18,568 (8096, 29999)	0.94 (0.42, 1.51)	811,530 (348312, 1312261)	37.81 (16.28, 60.02)	-0.12 (-0.4, 0.16)	0.35 (0.21, 0.49)
Eastern Europe	699 (289, 1052)	0.25 (0.11, 0.38)	37,778 (15614, 59516)	13.57 (5.58, 21.2)	3991 (1652, 6251)	1.08 (0.44, 1.70)	125,772 (52016, 198719)	34.64 (14.43, 54.83)	3.87 (2.29, 5.47)	2.65 (2.14, 3.17)
Eastern Sub-Saharan Africa	1011 (396, 1583)	1.74 (0.68, 2.71)	27,725 (10936, 43067)	39.94 (15.8, 61.87)	2101 (849, 3413)	1.67 (0.66, 2.75)	61,861 (24893, 100787)	39.73 (15.73, 64.62)	-0.33 (-0.4, -0.25)	-0.2 (-0.28, -0.12)
High-income Asia Pacific	1770 (755, 2741)	0.94 (0.40, 1.45)	87,131 (38199, 134072)	43.03 (18.8, 66.58)	2341 (974, 3752)	0.40 (0.17, 0.64)	220,368 (90908, 365666)	55.05 (23.1, 90.4)	-2.84 (-3.06, -2.63)	0.64 (0.52, 0.76)
High-income North America	4533 (1943, 7245)	1.22 (0.52, 1.95)	126,973 (53621, 202738)	35.35 (14.79, 56.31)	7010 (2838, 11170)	0.99 (0.40, 1.56)	377,333 (157838, 612359)	57.19 (24.1, 92.69)	-1.31 (-1.72, -0.9)	1.33 (1.19, 1.46)
North Africa and Middle East	3855 (1618, 5875)	2.91 (1.23, 4.45)	133,407 (57073, 205249)	83.27 (35.75, 128.41)	13,250 (5781, 20612)	3.56 (1.54, 5.50)	635,219 (275021, 1000668)	139.58 (60.62, 219.11)	1.1 (0.92, 1.29)	1.9 (1.8, 2)
Oceania	202 (81, 335)	7.93 (3.19, 12.92)	6848 (2768, 11133)	218.08 (88.15, 351.68)	567 (240, 934)	8.68 (3.61, 13.94)	21,957 (9024, 35300)	268.48 (110.53, 431.69)	0.23 (0.14, 0.32)	0.62 (0.53, 0.71)
South Asia	9411 (4011, 14611)	2.24 (0.95, 3.48)	287,342 (124713, 444683)	56.03 (24.35, 87.96)	38,490 (16586, 59222)	3.27 (1.41, 5.05)	1,125,155 (517606, 1779236)	82 (37.08, 128.77)	1.3 (1.14, 1.45)	1.17 (1.06, 1.27)
Southeast Asia	4848 (2035, 7482)	2.29 (0.96, 3.56)	152,373 (65089, 236779)	63.62 (27.37, 99.32)	16,808 (7385, 25418)	2.99 (1.32, 4.49)	592,992 (254841, 916715)	94.14 (40.25, 145.48)	0.79 (0.72, 0.87)	1.18 (1.14, 1.21)
Southern Latin America	548 (229, 897)	1.27 (0.54, 2.09)	14,798 (6126, 23921)	32.46 (13.5, 52.76)	830 (329, 1348)	0.91 (0.36, 1.47)	32,437 (13672, 52591)	36.76 (15.48, 59.81)	-1.14 (-1.34, -0.94)	0.26 (0.16, 0.35)
Southern Sub-Saharan Africa	1266 (555, 1951)	5.44 (2.42, 8.39)	34,916 (15217, 53106)	134.01 (59.27, 203.63)	4700 (1990, 7080)	9.89 (4.18, 15.08)	130,783 (56744, 195936)	239.25 (102.53, 361.94)	2.2 (1.76, 2.64)	2.09 (1.75, 2.43)
Tropical Latin America	2964 (1332, 4495)	3.90 (1.73, 5.91)	92,418 (41501, 146303)	106.25 (47.2, 164.62)	8338 (3435, 12846)	3.39 (1.40, 5.23)	276,105 (113987, 430442)	108.19 (44.79, 168.94)	-0.33 (-0.49, -0.17)	0.09 (0.03, 0.16)
Western Europe	10,492 (4478, 16245)	1.71 (0.73, 2.64)	245,675 (107620, 380381)	41.11 (18.08, 63.59)	11,492 (4875, 17852)	0.94 (0.40, 1.45)	388,561 (176058, 617043)	41.73 (18.57, 66.22)	-2 (-2.13, -1.86)	-0.12 (-0.21, -0.02)
Western Sub-Saharan Africa	1512 (587, 2413)	2.21 (0.86, 3.52)	42,567 (17194, 66326)	52.38 (20.99, 81.53)	4013 (1613, 6319)	2.77 (1.12, 4.36)	130,862 (53418, 206277)	71.51 (29.26, 112.18)	0.75 (0.69, 0.81)	1.01 (0.97, 1.04)

Generated from data available at http://ghdx.healthdata.org/gbd-results-tool

No., number; ASMR, age-standardized mortality rate; *UI*, uncertainty interval;

DALYs, disability-adjusted life years; ASDR, age-standardized DALY rate; EAPC, estimated annual percentage change; *CI*, confidence interval

### Regional variations and trends in T2DM burden attributed to LPA

**Fig. 1:**
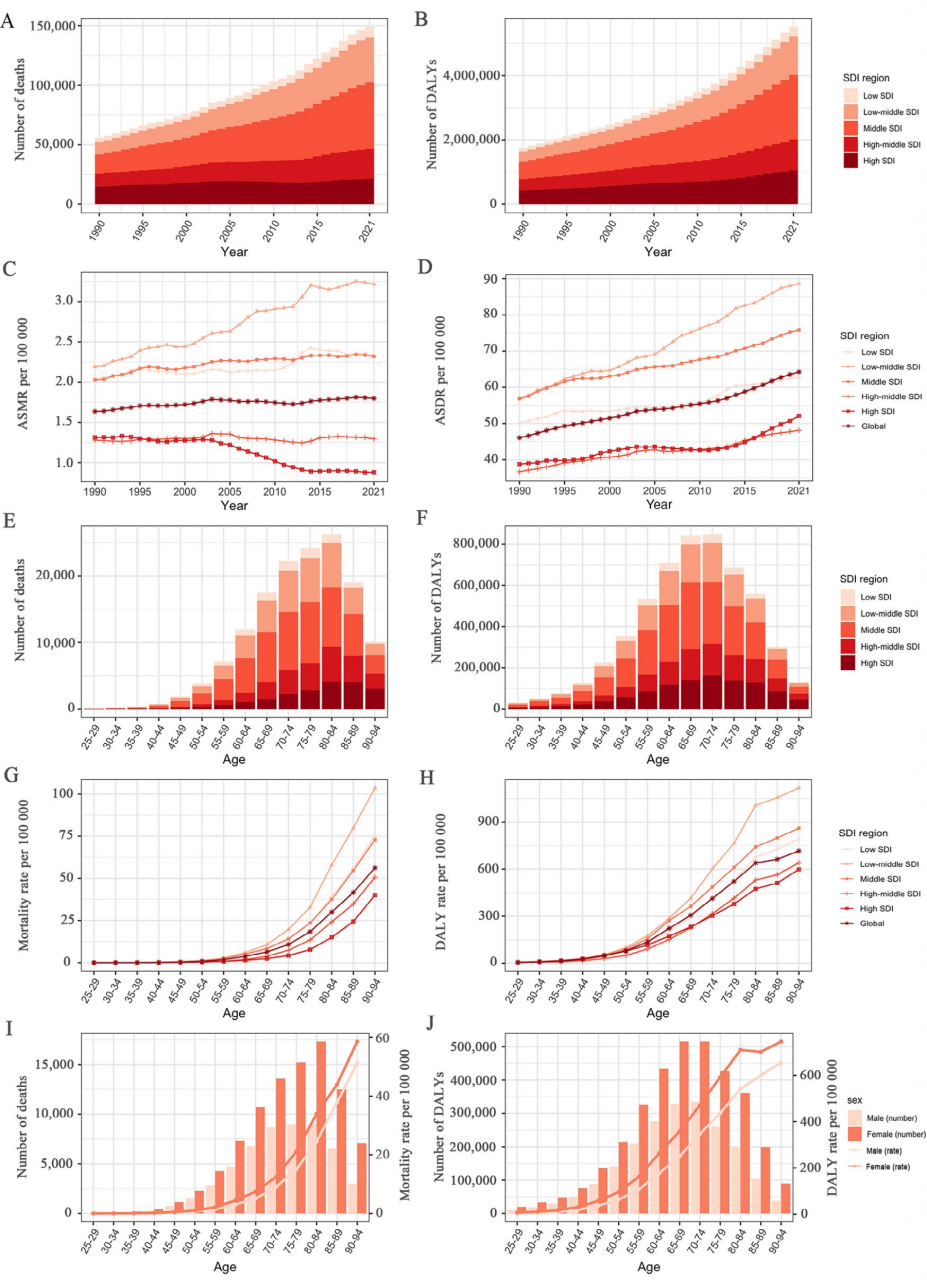
The T2DM burden attributable to low physical activity by SDI region. The global **(A)** deaths, **(B)** DALYs, **(C)** ASMR, and **(D)** ASDR of T2DM attributable to low physical activity for all ages from 1990 to 2021. The age distributions of **(E)** deaths and **(F)** DALYs, **(G)** age-specific mortality rate, and **(H)** age-specific DALY rate of T2DM attributable to LPA by age in 2021. **(I)** Deaths and age-specific mortality rate, and **(J)** DALY and age-specific DALY rate of T2DM attributable to low physical activity by sex in 2021. Generated from data available at http://ghdx.healthdata.org/gbd-results-tool. SDI, socio-demographic index; ASMR, age-standardized mortality rate; DALYs, disability-adjusted life years; ASDR, age-standardized DALY rate

At the SDI regional level, the greatest LPA-related T2DM deaths (0.06 million) and DALYs (2.01 million) were found in middle SDI regions, accounting for more than one-third of the total deaths and DALYs worldwide, whereas the lowest was found in low SDI regions (8,886 deaths and 0.30 million DALYs) in 2021 (Table 1; Fig. 1A-B). In 2021, the highest ASMR and ASDR were observed in low-middle SDI regions, while the lowest ASMR was observed in high SDI regions and the lowest ASDR was observed in high-middle SDI regions (Table 1; Fig. 1C-D). From 1990 to 2021, the ASMR in high SDI regions showed a downwards trend but exhibited an upwards trend in the other regions. The ASDR had substantially increased in all SDI regions, with EAPCs ranging from 0.68 (95% *CI*: 0.61, 0.74) to 1.46 (95% *CI*: 1.41, 1.50) (Table 1).

At the GBD regional level, the heaviest burden of T2DM attributable to LPA occurred in South Asia (38,490 deaths and 1.13 million DALYs), followed by East Asia (18,568 deaths and 0.81 million DALYs) and North Africa and the Middle East (13,250 deaths and 0.64 million DALYs) in 2021 (Table 1). In 2021, Southern Sub-Saharan Africa had the highest ASMR (9.89 per 100,000), while the lowest was observed in High-income Asia Pacific (0.40 per 100,000). In addition, Oceania had the highest ASDR (268.48 per 100,000), whereas Eastern Europe had the lowest (34.64 per 100,000). Among the regions, Eastern Europe had the fastest increase in both ASDR and ASMR, with EAPCs 3.87 (95% *CI*: 2.29, 5.47) and 2.65 (95% *CI*: 2.14, 3.17), respectively. High-income Asia-Pacific experienced the fastest decrease in ASMR (EAPC = -2.84, 95% *CI*: -3.06, -2.63), while Eastern Sub-Saharan Africa had the fastest decrease in ASDR (EAPC = -0.2, 95% *CI*: -0.28, -0.12) (Table 1).

**Fig. 2:**
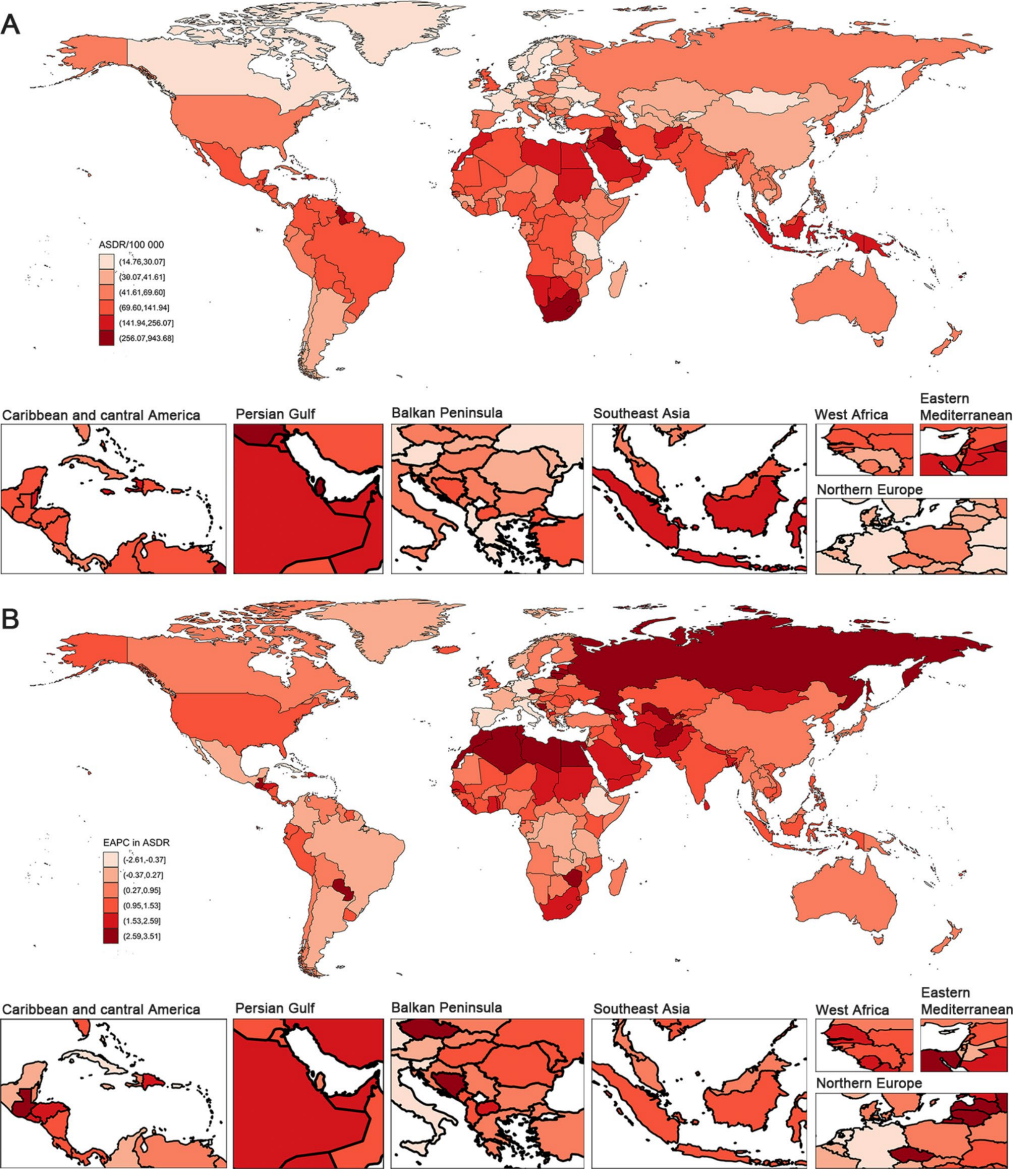
The spatial distribution of T2DM **(A)** ASDR and **(B)** the EAPC attributable to low physical activity in 2021. Generated from data available at http://ghdx.healthdata.org/gbd-results-tool. ASDR, age-standardized disability-adjusted life-years rate; EAPC, estimated annual percentage change

At the national level, India (31,152 deaths and 0.90 million DALYs), China (17,106 deaths and 0.76 million DALYs), and Indonesia (9,293 deaths and 0.34 million DALYs) were the top three T2DM deaths and DALYs attributable to LPA in 2021 (Supplementary Material 2 Table S1). In 2021, the Marshall Islands had the highest ASMR and ASDR (927.56 deaths and 943.68 DALYs per 100,000). In contrast, Ukraine recorded the lowest ASMR (0.16 deaths per 100,000) and Tanzania had the lowest ASDR (14.78 DALYs per 100,000) (Fig. 2; Supplementary Material 2 Figure S1). The Russian Federation showed the greatest increase in ASMR (EAPC = 4.65; 95% *CI*: 2.90, 6.44), while the Republic of Uzbekistan had the highest rise in ASDR (EAPC = 3.50; 95% *CI*: 3.17, 3.84). Singapore experienced the most significant decrease in ASMR (EAPC = -7.03; 95% *CI*: -8.09, -5.95), and Cyprus showed the fastest decline in ASDR (EAPC = -2.62; 95% *CI*: -2.74, -2.49) (Fig. 2; Supplementary Material 2 Figure S1 and Table S1).

### Age and sex variations in T2DM burden attributable to LPA

In 2021, the age-specific number of T2DM deaths attributable to LPA exhibited an inverted U-shaped relationship with age, peaking in the 80–84 age group; most deaths occurred in the 65–89 age range, with a higher number in females compared to males (Fig. 1E and I). Correspondingly, the age-specific mortality rate increased with age globally and persistently remained higher in females than in males (Fig. 1G and I). The age-specific number of T2DM DALYs attributable to LPA followed a similar pattern to deaths, but the peak point appeared in the 65–69 age group, with a higher number of DALYs occurring in the 55–84 age group (Fig. 1F). Likewise, the age-specific DALY rate increased with age across all SDI regions, and consistently remained higher in females than in males (Fig. 1H and J).

**Fig. 3:**
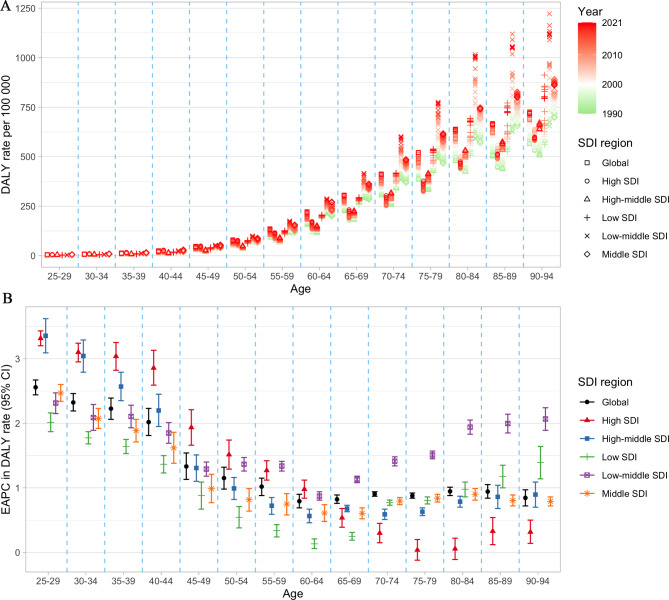
The age distribution of **(A)** age-specific DALY rate and **(B)** EAPC in age-specific DALY rate attributable to LPA by SDI region from 1990 to 2021. Generated from data available at http://ghdx.healthdata.org/gbd-results-tool. DALY, disability-adjusted life year; EAPC, estimated annual percentage change; LPA, low physical activity; SDI, Socio-demographic Index

**Fig. 4:**
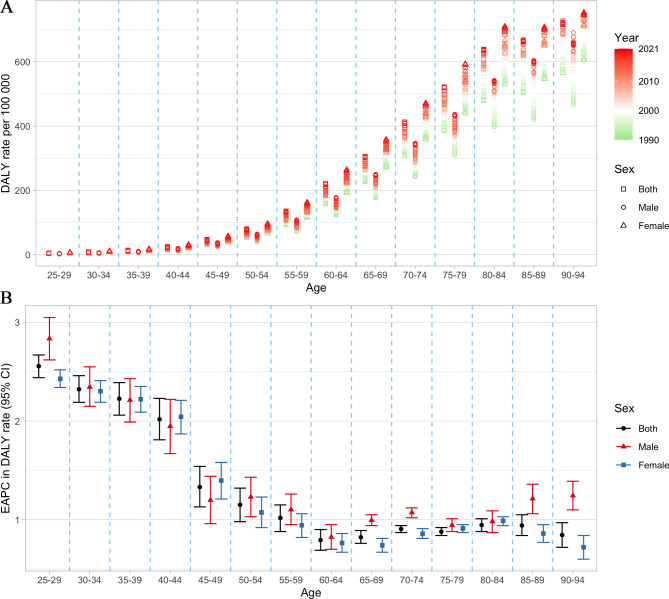
The age distribution of **(A)** age-specific DALY rate and **(B)** EAPC in age-specific DALY rate attributable to LPA by sex from 1990 to 2021. Generated from data available at http://ghdx.healthdata.org/gbd-results-tool. DALY, disability-adjusted life year; EAPC, estimated annual percentage change; LPA, low physical activity

As age increases, the age-specific mortality rate increased across all SDI regions (Supplementary Material 2 Figure S2). The EAPC was greater than 0 across all age groups in low-middle SDI regions, indicating that mortality rates were increasing across all age groups. In contrast, in high SDI regions, the EAPC was generally less than 0 after the 45–49 age groups, suggesting that mortality rates were decreasing in these age groups. A similar pattern was observed in the DALY rate, while the EAPC was greater than 0 across all age groups in all SDI regions (Fig. 3). Globally, the age-specific mortality rate nearly increased in males across all age groups, with the most rapid increases observed in the 25–29 age group. In females, the age-specific mortality rate increased in the 25–29, 35–44 and 75–94 age groups, with the fastest increase occurring in the 40–44 age group (Supplementary Material 2 Figure S3). The age-specific DALY rate showed increasing trends globally, with the 25–29 age group experiencing the greatest increase (Fig. 4). This pattern remained consistent for both females and males.

### T2DM burden attributable to LPA associated with SDI

**Fig. 5:**
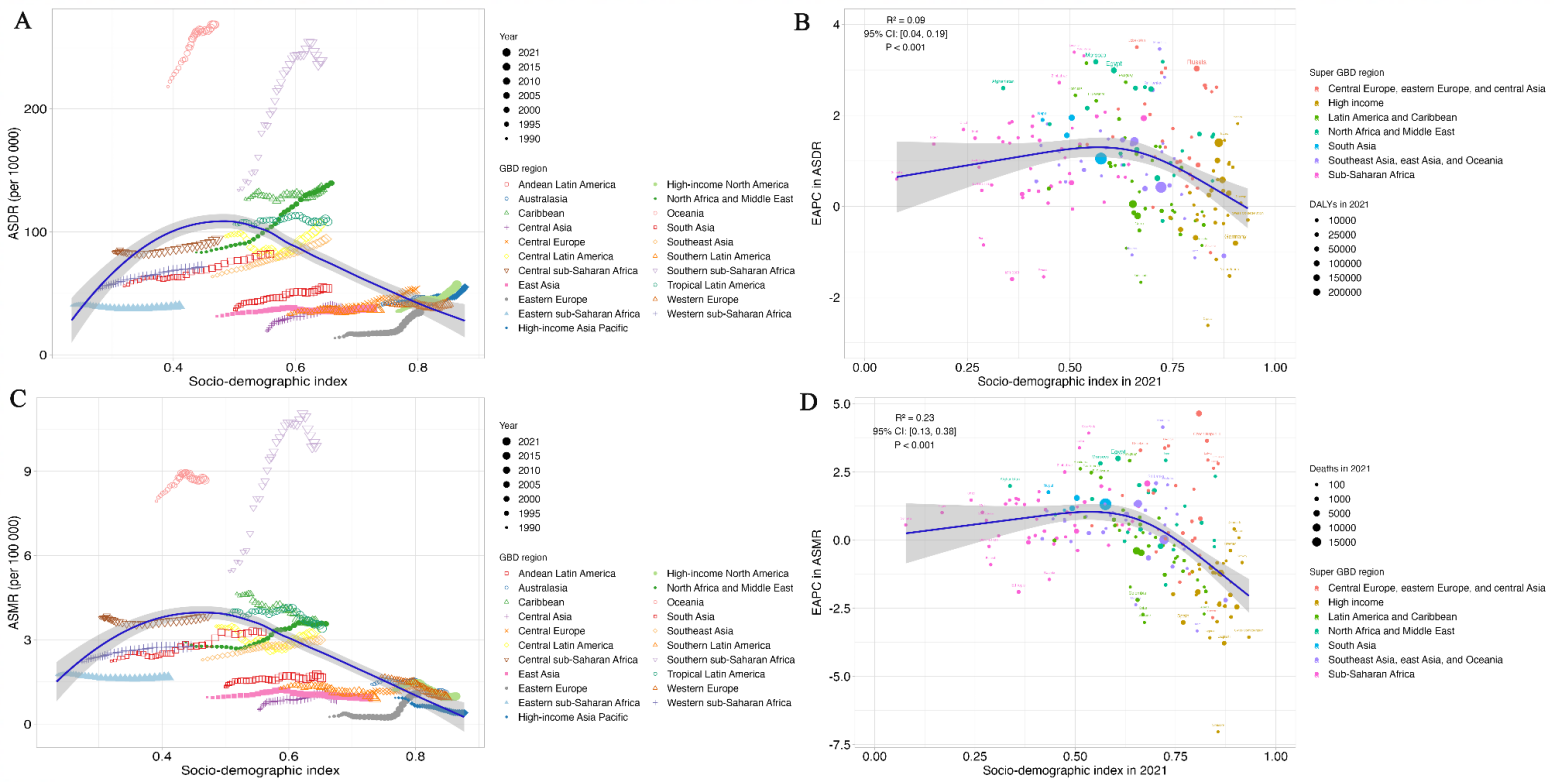
The relationship between T2DM burden attributable to low physical activity **(A)** ASDR and SDI from 1990 to 2021 by GBD region, **(B)** EAPC in ASDR and SDI in 2021 by super GBD region, and **(C)** ASMR and SDI from 1990 to 2021 by GBD region, **(D)** EAPC in ASMR and SDI in 2021 by super GBD region. Generated from data available at http://ghdx.healthdata.org/gbd-results-tool. ASDR, age-standardized disability-adjusted life-year rate; ASMR, age-standardized mortality rate; SDI, Socio-demographic Index; EAPC, estimated annual percentage change

In general, the ASDR and ASMR of LPA-related T2DM exhibited an inverted U-shaped association with the SDI, with the peak occurring at an SDI value of approximately 0.5 (Fig. 5A and C). This finding suggests that regions with low-middle and middle SDI bore the highest burden of LPA-related T2DM. Similarly, the EAPC in the ASDR and ASMR also displayed an inverted U-shaped association with the SDI in 2021 (Fig. 5B and D). The fitted line indicated that the largest increase in the burden of T2DM associated with LPA occurred in low-middle and middle SDI countries.

## Discussion

In this study, spatiotemporal trends in deaths and DALYs of T2DM attributable to LPA were estimated in 204 countries and territories. Over the last 32 years, the global age-standardized rates of mortality and DALYs of LPA-related T2DM have increased by 9.8% and 39.5%, respectively, with the total number more than tripled. The burden of T2DM attributable to LPA varied considerably across the world, with the highest ASDR occurring in Oceania, Southern Sub-Saharan Africa, and North Africa and Middle East, whereas Eastern Europe, Southern Sub-Saharan Africa, and Central Asia ​experienced the largest increases between 1990 and 2021. The low-middle SDI regions exhibited the highest rates of DALYs for LPA-related T2DM in 2021 and experienced the most significant increase from 1990 to 2021. The ASMR or ASDR had a reverse U-shaped relationship with SDI, with the most severe burden observed in the low-middle and middle SDI regions. The age group older than 60 years had the highest rate of DALYs for LPA-related T2DM in 2021, while the age group 25–44 years showed the largest increase between 1990 and 2021.

The most recent analysis from GBD 2021 highlights a troubling global trend: the prevalence of T2DM is persistently escalating across all countries, territories, age groups, and genders [1]. In 2021, the global age-standardized prevalence of diabetes stood at 6.1%. Notably, the highest rates were recorded in North Africa and the Middle East, reaching 9.3%, and in the Oceania region, where it peaked at 12.3%^1^. The global surge in T2DM is intricately linked to the demographic ageing transition, with an increasingly ageing population contributing significantly to the rising prevalence [20]. Additionally, the interconnected epidemics of obesity and physical inactivity significantly contribute to exacerbating the T2DM epidemic to unprecedented levels. Physical activity emerges as a critical determinant in this complex web of factors, playing a pivotal role in weight maintenance, enhancing insulin sensitivity, and regulating blood sugar levels. Increasingly robust evidence spanning various populations and age groups corroborates the hypothesis that sufficient physical activity correlates with a reduced risk of T2DM, whereas physical inactivity significantly contributes to its rising prevalence [21, 22]. A meta-analysis revealed a dose-response relationship, indicating that the risk of T2DM decreased by 15% with each increment of 20 MET-hours per week in leisure-time activity [23]. However, despite the well-established benefits of physical activity, our results indicated the proportion of LPA cases did not improve globally from 1990 to 2021. Following its designation as a global pandemic in the 2012 Lancet Series, physical inactivity has prompted urgent calls for action across all sectors of government and society. Subsequent to this recognition, advancements have been noted in various domains, including expanded national surveillance, policy adoption, and research on correlates and interventions in the majority of countries [11]. However, significant challenges in implementation persist. According to the WHO, around a quarter of global national policies pertaining to physical activity were not implemented as of 2015 [24]. This substantial implementation gap underscores the difficulties countries face in translating policy intentions into concrete actions.

### Regional disparities

A wide range of geographical variations was found in the LPA-related T2DM burden, with both the ASMR and ASDR being greater in regions with the lowest SDI than in those with the highest SDI. The measure of SDI, which combines factors such as income, education, and fertility rates, may be associated with these differences, as these are key determinants of health outcomes across populations. These variations in the burden of T2DM attributable to LPA between lower and higher SDI indicate societal spatial inequalities in T2DM prevention, healthcare, and physical activity. In lower SDI countries, inadequate infrastructure and capacity, exorbitant medication costs, fragmented healthcare systems, health illiteracy, and social disparities contribute to a significant number of individuals with T2DM going undiagnosed, untreated, or poorly managed, leading to the development of acute and chronic complications, and premature mortality [20]. In 2010, despite 70% of T2DM patients residing in lower SDI countries, more than 90% of the global expenditure on diabetes care occurred in higher SDI countries [25]. Individuals with diabetes in SDI countries endure premature mortality at a younger and more productive age compared to their counterparts in higher SDI countries [20]. Regions like North Africa and the Middle East, Central Latin America, and Oceania had the highest age-standardized years lived with disability (YLD) rates for complications related to diabetes, signifying the most affected areas [26]. Conversely, the occurrence of diabetes-related complications has declined over recent decades in affluent regions like North America, Europe, and Australia [27, 28]. Higher health literacy and better accessibility to healthcare contribute to a reduction in mortality rates among T2DM patients due to complications. This observation may elucidate why, despite higher prevalence of physical inactivity in high SDI regions compared to low SDI regions, the ASDR attributed to T2DM related to LPA are lower in high SDI regions than in low SDI regions.

The association between the global burden of T2DM attributable to LPA and the SDI was not monotonic, but showed an inverted U-shape, with low-middle and middle SDI regions experiencing the heaviest burden. This can be attributed to the combined effects of a high prevalence of physical inactivity and inadequate healthcare. For example, in Oceania, countries like Fiji and other Pacific Islands have seen significant increases in non-communicable diseases (NCDs), such as T2DM, driven by rising obesity rates and declining physical activity levels [29]. Fiji has one of the highest global obesity rates, with 33% of adults in the Pacific region overweight or obese as of 2014 [29, 30]. The lack of physical activity, compounded by environmental and societal factors, has exacerbated the obese trends. Indigenous populations face particularly severe impacts due to historical marginalization, limited access to healthcare, and socio-economic disparities [30]. These macro- and micro-level factors have significantly shaped their health outcomes [31, 32]. In Southern Sub-Saharan Africa, rapid urbanization has similarly led to declining physical activity and rising T2DM prevalence. South Africa, for instance, has one of the highest rates of physical inactivity globally, with 43–49% of individuals aged 15 and older engaging in minimal or no physical activity [33]. The transition from agriculturally-based economies to cash-based economies has reduced the need for physical labor, shifting occupational and transportation-related activities towards more sedentary behaviors [33]. Moreover, inadequate healthcare coverage exacerbates the problem. According to the NCD Risk Factor Collaboration, fewer than 4 out of 10 adults with diabetes in South Africa receive glucose-lowering medications [34]. In the GBD study, health care system performance is evaluated using a ratio that compares the actual disease burden to the expected disease burden [35]. A ratio exceeding 1 signifies that the actual burden surpasses the anticipated burden, suggesting suboptimal healthcare system performance. In both Southern Sub-Saharan Africa and Oceania, the observed YLDs attributed to diabetes were nearly double the anticipated levels, suggesting inadequacies in the quality of local healthcare to address the needs of the diabetic population [35].

Interestingly, low SDI regions bear a lighter burden of LPA-related T2DM compared to low-middle and middle SDI regions. This could be due to higher physical exertion in occupational and daily activities or underreporting of T2DM and physical activity data due to limited healthcare and surveillance systems [32, 36]. In contrast, high SDI regions show declining mortality from advanced healthcare systems but increasing DALYs from LPA-related T2DM, emphasizing the need for preventive strategies like promoting physical activity to mitigate this growing burden [11].

### Gender disparities and age-related trends

The global trends in physical activity indicate that younger people and males are more inclined to engage in physical activity [36]. Prior studies have indicated that inactivity increases with advancing age, a trend rooted in robust biological foundations [37]. A reduction in dopamine release and dopamine receptor activity is a significant factor associated with an age-related decrease in physical activity [37]. Leisure-time physical activity is often less frequent and less intense in females than in males [38]. This physical activity trend aligns with our study findings; Our findings revealed that the age-specific rates of mortality and DALYs attributable to LPA-related T2DM surged with increasing age across both genders. The most substantial burden was noted in individuals aged 60 years and above. Ageing, poor nutritional status, and physical inactivity are major drivers of T2DM [20]. Future health promotion initiatives and public health policies might necessitate a heightened emphasis on advocating for moderate physical activity among the elderly population. Nonetheless, the predicament also warrants attention for younger age cohorts. We observed that, among those aged 25–44 years, the burden of LPA-related T2DM showed the most significant increase. This may reflect changes in contemporary lifestyles, including increased sedentary behavior, the prevalence of convenience foods, and the rise of digital entertainment [11]. This trend not only highlights the health challenges faced by the younger generation but also emphasizes the need for targeted guidance and encouragement for younger individuals to adopt healthier lifestyles. Like in terms of male and female physical activity, sex differences existed in the burden of LPA-related T2DM, which was greater among females than in males globally. Mielke et al. reported that sex disparities in the prevalence of physical inactivity exhibit significant variability, regardless of the country’s wealth [38]. Although the causes for this gender variation are not yet clear, cultural influence, traditional roles, and lack of social support may be important reasons why females reduce their participation in physical activity [32]. Consequently, offering females increased access to safe and easily accessible leisure-time pursuits can serve to elevate their overall activity levels, thereby contributing to narrowing the gender disparity.

### Future directions

Our research findings revealed a notable escalation in the global burden of LPA-related T2DM from 1990 to 2021, with a particularly marked rise observed in regions categorized as low-middle and middle on the SDI scale. Ongoing economic development and societal transitions portend a probable surge in the prevalence of physical inactivity and T2DM in low-middle and middle SDI regions in the foreseeable future. To effectively reduce the global burden of T2DM, strong intervention measures promoting physical activity must be implemented. The 2012, 2016 and 2021 Lancet series on physical activity provided a range of evidence-based intervention strategies [11, 24, 39, 40]. These approaches highlight the significance of encouraging physical activity across various dimensions—spanning personal aspects (e.g., biological and psychological factors), social interactions (e.g., family and peers), and environmental constructs (e.g., communities equipped with parks and bicycle amenities) [11]. Given that the policies obstructing physical activity originate from the realms of transportation, education, sports, recreation, and urban planning sectors, intersectoral collaboration emerges as imperative within these strategies [24, 39]. Furthermore, exploring and leveraging innovative approaches, such as digital health and technology-based tools, is vital for fostering and monitoring physical activity [40]. Overall, these strategies provide us with a clear framework in which combining diverse approaches is necessary to promote physical activity.

### Study strengths and limitations

To our understanding, this research is the most exhaustive and current analysis delineating temporal and spatial patterns in the burden of T2DM attributed to LPA, examining variations across years, age groups, geographic locations, genders, and SDI categories. This analysis is based on the latest GBD 2021 dataset. Nevertheless, it’s crucial to acknowledge several limitations inherent in this study, which stem from the constraints of the GBD 2021 study. First, the precision of estimates regarding the burden of T2DM attributable to LPA primarily hinges on the accessibility of high-quality and standardized data sources. Nonetheless, data were not uniformly accessible for every country or year, particularly in certain low SDI regions like sub-Saharan Africa. The lack of comprehensive data in low SDI regions may contribute to the underestimation of the actual T2DM burden related to LPA. Consequently, prudence is warranted when interpreting the results, especially in countries exhibiting wider 95% UIs. Second, it is essential to acknowledge the limitations inherent in the currently available data on physical activity, primarily because these data rely on self-reported questionnaires, which are prone to recall bias and social desirability bias. Moreover, unmeasured confounders, such as genetic factors, diet, and environmental influences, may not be fully accounted for, potentially confounding the observed relationship between physical inactivity and T2DM. Fourth, while the GBD Collaborator has made substantial efforts to minimize misclassification bias, we acknowledge that some degree of underreporting or misclassification may still exist. Fifth, The GBD 2021 ranked LPA 18th out of 20 risk factors for DALYs, down from 10th in the 2010 GBD publication [41]. Attributable deaths decreased from 3.2 million in 2010 to approximately 0.66 million in 2021, both significantly lower than the Lancet 2012 physical activity series estimate of 5 million deaths per year [42]. In addition, Previous studies reported that the prevalence of insufficient physical activity has generally risen globally over the past few decades [24, 25]. Therefore, the GBD 2021 study may underestimate the actual T2DM burden attributable to LPA, especially in the context of aging and increased life expectancy worldwide. Sixth, physical activity is a multidimensional behavior that affects health through the frequency, duration, intensity, and activity domains. The GBD 2021 study only used a single indicator-total metabolic equivalence to describe physical activity. Enhancements in the methodologies employed for capturing and categorizing physical activity are imperative in forthcoming iterations of the GBD study. This would facilitate a more comprehensive estimation of the overall burdens attributable to LPA. Finally, it’s worth noting that in the GBD 2021 study, the theoretical minimum-risk exposure level for physical inactivity was set at 3000–4500 MET-minutes per week. This threshold is five times higher than the minimum amount of physical activity recommended by the WHO to attain any health benefit, which stands at 600 MET-minutes per week. These findings seem to deviate substantially from established thresholds for minimal physical activity, although GBD collaborators explain that they aim to enhance the accuracy of capturing any additional protective effects stemming from higher activity levels.

### Public health implications

Substantial increases in national action are urgently needed to target populations disproportionately affected by LPA. It is recommended that future actions be adapted and implemented based on the Global Action Plan on Physical Activity 2018–2030 and the WHO 2020 guidelines on physical activity and sedentary behavior, as well as local contexts in all countries [43, 44]. Successful examples from some countries provide actionable strategies, such as community walking groups in rural India [39, 45], physical activity classes in Brazil [46], and community-wide policies in Latin America to improve built environments and promote physical activity [39]. The findings from this study call for health ministries and countries across the globe that provide greater resources for the promotion of physical activity through active transport, active recreation, active sport, and active living. In particular, we should focus on children and adolescents, offering more opportunities for physical activity and ensuring a healthier future for them than for their parents.

## Conclusions

Over the past 32 years, the global burden of T2DM attributable to LPA has continued to increase at an alarming rate in almost all countries, particularly in low-middle and middle SDI regions. The burden of T2DM attributable to LPA is greater in females, older than 60 years of age groups, and in developing countries.

## Electronic supplementary material

Below is the link to the electronic supplementary material.

Supplementary Material 1

Supplementary Material 2

## Data Availability

All the data are available in the GBD Data Tool repository (http://ghdx.healthdata.org/gbd-results-tool).

## Abbreviations

ASDR: Age-standardized disability-adjusted life-year rate
ASMR: Age-standardized mortality rate
DALYs: Disability-adjusted life years
EAPC: Estimated annual percentage change
GBD: Global Burden of Disease Study
LPA: Low physical activity
MET: Metabolic equivalent
SDI: Socio-demographic index
STROBE: Strengthening the Reporting of Observational Studies in Epidemiology
UI: Uncertainty interval
