# Supplementary material for: The global, regional, and national burden of type 2 diabetes mellitus attributable to low physical activity from 1990 to 2021: a systematic analysis of the global burden of disease study 2021
Source: Int J Behav Nutr Phys Act. 2025 Jan 16;22:8. doi: 10.1186/s12966-025-01709-8 (PMC11740459; doi:10.1186/s12966-025-01709-8)
Supplement: Supplementary file 2 — Supplementary Material 2 [file 12966_2025_1709_MOESM2_ESM.docx]

**Appendix. Supplementary data**

**Supplementary Figure S1** The spatial distribution of T2DM **(A)** ASMR and **(B)** the EAPC attributable to LPA in 2021. T2DM, type 2 diabetes mellitus; ASMR, age-standardized mortality rate; EAPC, estimated annual percentage change; LPA, low physical activity.

**Supplementary Figure S2** The age distribution of **(A)** age-specific mortality rate and **(B)** EAPC in age-specific mortality rate attributable to LPA by SDI region from 1990 to 2021. EAPC, estimated annual percentage change; LPA, low physical activity; SDI, Socio-demographic Index.

**Supplementary Figure S3** The age distribution of **(A)** age-specific mortality rate and **(B)** EAPC in age-specific mortality rate attributable to LPA by sex from 1990 to 2021. EAPC, estimated annual percentage change; LPA, low physical activity.

**Supplementary Table S1** The global T2DM burden attributable to low physical activity in 204 countries and territories between 1990 and 2021.

**Supplementary Figure S1** The spatial distribution of T2DM **(A)** ASMR and **(B)** the EAPC attributable to LPA in 2021. T2DM, type 2 diabetes mellitus; ASMR, age-standardized mortality rate; EAPC, estimated annual percentage change; LPA, low physical activity.


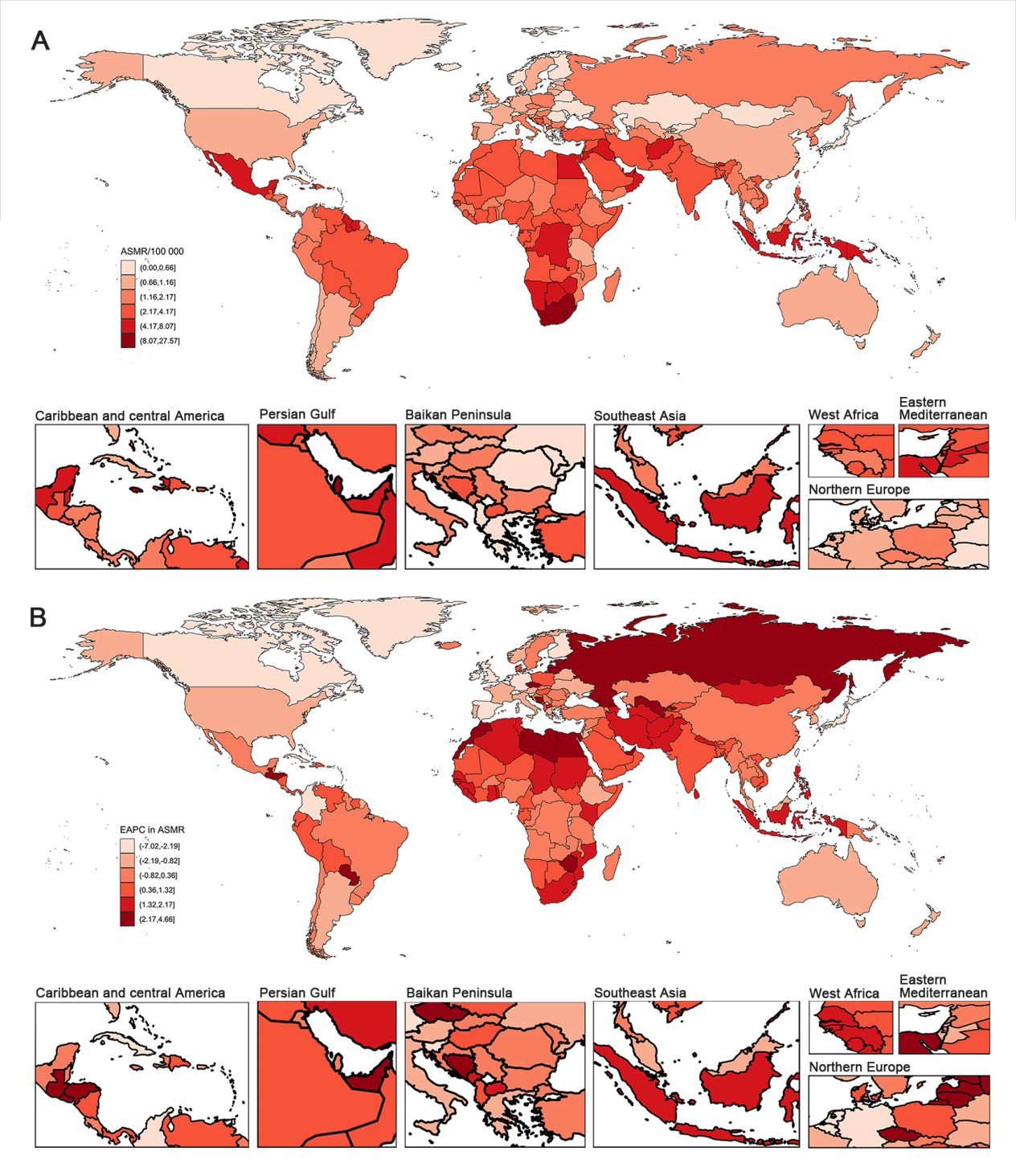


**Supplementary Figure S2** The age distribution of **(A)** age-specific mortality rate and **(B)** EAPC in age-specific mortality rate attributable to LPA by SDI region from 1990 to 2021. EAPC, estimated annual percentage change; LPA, low physical activity; SDI, Socio-demographic Index.


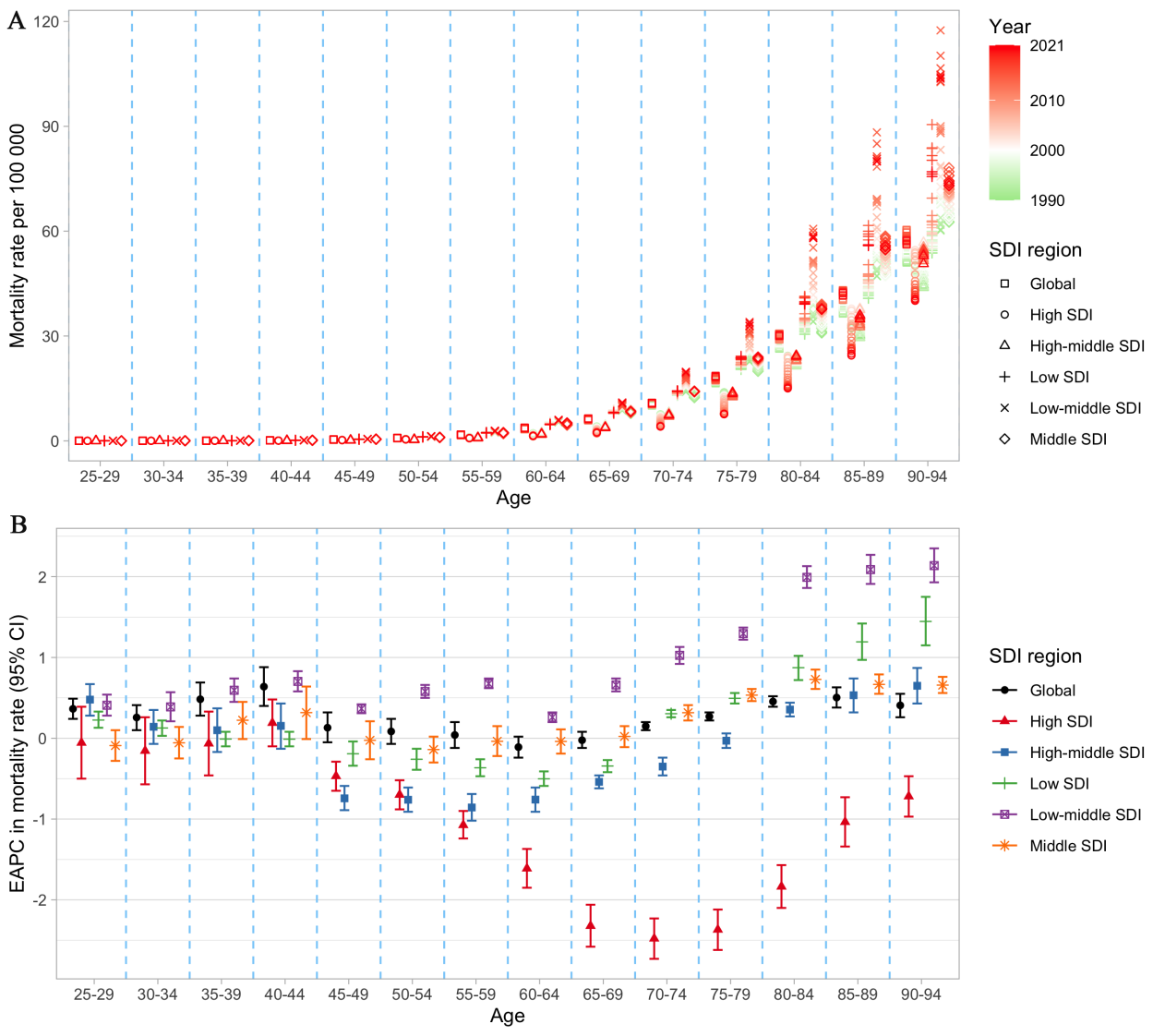


**Supplementary Figure S3** The age distribution of **(A)** age-specific mortality rate and **(B)** EAPC in age-specific mortality rate attributable to LPA by sex from 1990 to 2021. EAPC, estimated annual percentage change; LPA, low physical activity.


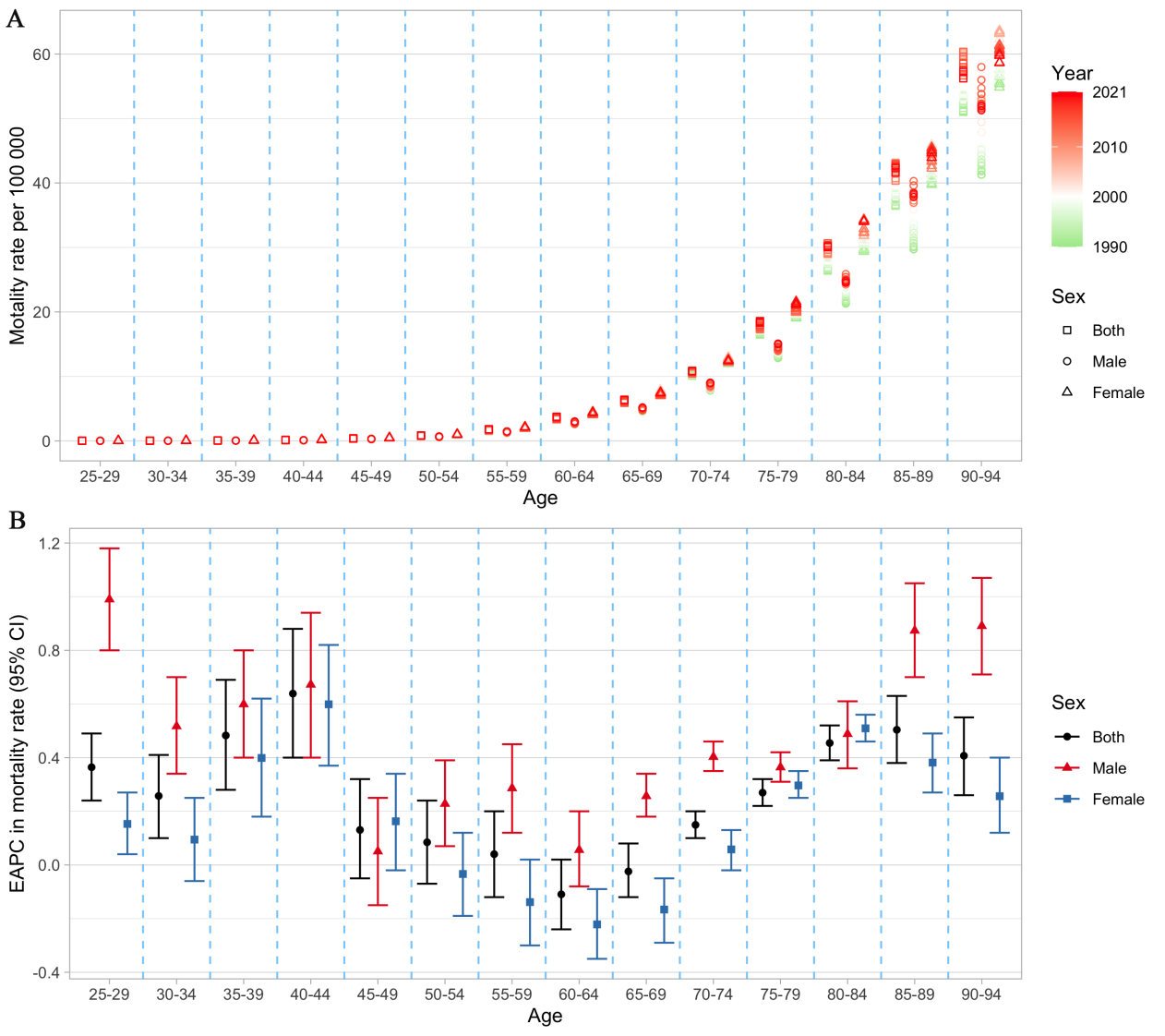


**Supplementary Table S2** The global T2DM burden attributable to low physical activity in 204 countries and territories between 1990 and 2021

| **Characteristics** | **1990** | | | | |  | **2021** | | | |  | **EAPC (1990-2021)** | |
| --- | --- | --- | --- | --- | --- | --- | --- | --- | --- | --- | --- | --- | --- |
|  | **Deaths cases No.(95%UI)** | | **ASMR per 10^5^ No.(95%UI)** | **DALYs**  **No.(95%UI)** | **ASDR per 10^5^ No.(95%UI)** |  | **Deaths cases No.(95%UI)** | **ASMR per 10^5^ No.(95%UI)** | **DALYs**  **No.(95%UI)** | **ASDR per 10^5^ No.(95%UI)** |  | **ASMR**  **No.(95%CI)** | **ASDR**  **No.(95%CI)** |
| American Samoa | 3 (1, 4) | 12.76 (5.54, 19.86) | | 98 (45, 153) | 397.62 (182.24, 619.83) |  | 7 (3, 11) | 15.68 (6.92, 25.65) | 305 (135, 478) | 597.82 (266.75, 940.86) |  | 0.56 (0.19, 0.93) | 1.2 (0.89, 1.5) |
| Antigua and Barbuda | 4 (2, 6) | 6.37 (2.66, 10.03) | | 87 (37, 138) | 159.08 (68.29, 250.64) |  | 6 (2, 9) | 5.9 (2.42, 9.39) | 182 (72, 293) | 174.01 (68.99, 280.33) |  | -0.67 (-0.88, -0.46) | -0.09 (-0.24, 0.05) |
| Arab Republic of Egypt | 669 (274, 1005) | 3.3 (1.41, 5.08) | | 21187 (8852, 32402) | 81.01 (34.03, 125.46) |  | 2719 (1163, 4411) | 6.12 (2.67, 9.88) | 101561 (43387, 164081) | 171.11 (74.75, 275.47) |  | 3 (2.61, 3.39) | 2.99 (2.76, 3.22) |
| Argentine Republic | 406 (165, 680) | 1.35 (0.56, 2.28) | | 10496 (4234, 17233) | 33.05 (13.48, 54.16) |  | 555 (229, 902) | 0.95 (0.39, 1.54) | 19640 (7918, 32991) | 34.59 (13.97, 58.6) |  | -1.27 (-1.47, -1.08) | -0.1 (-0.23, 0.04) |
| Australia | 281 (118, 434) | 1.49 (0.61, 2.29) | | 7979 (3559, 12766) | 40.77 (17.98, 65.2) |  | 582 (250, 927) | 1.12 (0.48, 1.76) | 21436 (9320, 35012) | 48 (21.05, 77.82) |  | -1.19 (-1.51, -0.88) | 0.35 (0.25, 0.45) |
| Barbados | 32 (15, 49) | 10.44 (4.69, 15.9) | | 773 (350, 1194) | 265.62 (120.13, 410.25) |  | 44 (19, 71) | 8.4 (3.68, 13.56) | 1300 (593, 2007) | 255.6 (116.51, 393.18) |  | -0.81 (-1.08, -0.54) | -0.27 (-0.42, -0.12) |
| Belize | 4 (2, 6) | 4.56 (1.88, 7.1) | | 109 (46, 174) | 118.61 (50.19, 188.81) |  | 14 (6, 22) | 5.11 (2.09, 8.2) | 470 (202, 762) | 158.01 (67.26, 255.22) |  | 0.36 (-0.21, 0.93) | 0.9 (0.5, 1.31) |
| Bermuda | 2 (1, 3) | 3.59 (1.57, 5.65) | | 52 (23, 81) | 85.57 (38.41, 133.4) |  | 3 (1, 4) | 1.66 (0.74, 2.62) | 94 (40, 154) | 68.61 (29.59, 112.36) |  | -2.71 (-2.84, -2.57) | -0.86 (-1.01, -0.71) |
| Bolivarian Republic of Venezuela | 189 (75, 309) | 2.23 (0.89, 3.65) | | 5715 (2321, 9384) | 61.1 (24.76, 99.91) |  | 809 (334, 1345) | 2.92 (1.21, 4.88) | 25006 (10681, 41554) | 84.35 (36.42, 139.47) |  | 0.75 (0.4, 1.1) | 0.95 (0.71, 1.19) |
| Bosnia and Herzegovina | 40 (17, 66) | 1.17 (0.49, 1.95) | | 1514 (648, 2504) | 39.35 (17.09, 63.68) |  | 178 (73, 301) | 2.71 (1.11, 4.53) | 5485 (2344, 8963) | 84.75 (35.83, 137.93) |  | 3.38 (3.05, 3.72) | 2.94 (2.69, 3.2) |
| Brunei Darussalam | 7 (3, 11) | 8.4 (3.42, 13.87) | | 187 (75, 304) | 194.85 (78.88, 319.12) |  | 13 (5, 21) | 5.37 (2.16, 8.6) | 633 (270, 1066) | 182.25 (74.92, 302.7) |  | -1.07 (-1.28, -0.86) | -0.18 (-0.24, -0.11) |
| Burkina Faso | 59 (23, 99) | 1.82 (0.68, 3.11) | | 1574 (639, 2595) | 40.49 (16.51, 66.68) |  | 120 (46, 203) | 1.74 (0.65, 3.01) | 3893 (1519, 6651) | 46.24 (17.71, 79.03) |  | -0.23 (-0.3, -0.16) | 0.35 (0.27, 0.43) |
| Canada | 267 (98, 471) | 0.84 (0.31, 1.48) | | 5709 (2065, 9622) | 17.45 (6.36, 29.21) |  | 406 (153, 721) | 0.48 (0.18, 0.85) | 17588 (6490, 32057) | 23.3 (8.72, 42.59) |  | -2.42 (-3.04, -1.79) | 0.34 (0.07, 0.61) |
| Central African Republic | 24 (10, 40) | 2.85 (1.13, 4.96) | | 769 (317, 1275) | 71.47 (29.36, 117.96) |  | 46 (17, 84) | 3.01 (1.14, 5.6) | 1809 (695, 3129) | 84.24 (34.63, 149.11) |  | 0.16 (0.08, 0.24) | 0.47 (0.41, 0.54) |
| Commonwealth of Dominica | 4 (1, 6) | 6.00 (2.4, 9.84) | | 82 (34, 132) | 137.2 (56.52, 221.38) |  | 5 (2, 8) | 6.21 (2.53, 10.28) | 122 (49, 198) | 151.58 (60.71, 243.13) |  | -0.19 (-0.32, -0.07) | 0.04 (-0.06, 0.13) |
| Commonwealth of the Bahamas | 7 (3, 10) | 4.74 (2.06, 7.49) | | 200 (86, 327) | 132.45 (58.08, 217.17) |  | 12 (5, 20) | 3.49 (1.41, 5.71) | 529 (221, 873) | 131.19 (55.13, 214.8) |  | -1.46 (-1.73, -1.18) | -0.32 (-0.47, -0.16) |
| Cook Islands | 1 (1, 2) | 12.11 (4.81, 19.61) | | 41 (16, 66) | 325.3 (126.54, 517.65) |  | 2 (1, 4) | 9.35 (3.9, 15.64) | 80 (35, 130) | 314.89 (134.86, 510.58) |  | -1.04 (-1.17, -0.91) | -0.27 (-0.35, -0.19) |
| Czech Republic | 188 (74, 300) | 1.34 (0.53, 2.15) | | 5993 (2519, 9796) | 42.38 (17.86, 69.9) |  | 510 (216, 819) | 2.11 (0.89, 3.37) | 15749 (7088, 25437) | 69.12 (30.67, 111.33) |  | 3.65 (2.7, 4.61) | 2.66 (2.22, 3.09) |
| Democratic People's Republic of Korea | 149 (57, 267) | 1.18 (0.45, 2.13) | | 5281 (2003, 9045) | 35.13 (13.76, 61.33) |  | 373 (146, 635) | 1.22 (0.47, 2.08) | 15700 (6644, 26795) | 48.27 (20.5, 83.03) |  | 0.29 (0.11, 0.47) | 1.06 (0.98, 1.15) |
| Democratic Republic of Sao Tome and Principe | 0 (0, 1) | 0.53 (0.21, 0.94) | | 11 (5, 18) | 17.56 (7.45, 29.75) |  | 1 (0, 1) | 0.76 (0.29, 1.31) | 32 (12, 53) | 30.21 (11.78, 49.45) |  | 1.22 (1.1, 1.34) | 1.72 (1.65, 1.79) |
| Democratic Republic of the Congo | 434 (164, 732) | 4.25 (1.67, 7.08) | | 11692 (4411, 19750) | 90.43 (34.39, 148.79) |  | 1037 (402, 1792) | 4.24 (1.77, 7.24) | 31170 (12477, 53939) | 99.85 (41.59, 169.4) |  | -0.08 (-0.21, 0.05) | 0.27 (0.14, 0.4) |
| Democratic Republic of Timor-Leste | 2 (1, 3) | 0.74 (0.28, 1.32) | | 50 (20, 88) | 19.88 (7.68, 34.08) |  | 7 (2, 12) | 0.91 (0.31, 1.65) | 278 (107, 490) | 33.81 (12.55, 59.05) |  | 0.78 (0.58, 0.98) | 1.81 (1.65, 1.98) |
| Democratic Socialist Republic of Sri Lanka | 122 (44, 208) | 1.48 (0.54, 2.62) | | 3868 (1398, 6778) | 40.15 (14.81, 69.44) |  | 536 (201, 957) | 2.18 (0.82, 3.83) | 20342 (7847, 34901) | 75.52 (29.45, 126.08) |  | 2.09 (1.78, 2.41) | 2.56 (2.37, 2.76) |
| Dominican Republic | 65 (26, 103) | 2.11 (0.84, 3.36) | | 2427 (974, 3862) | 67.03 (27.41, 106.22) |  | 248 (106, 410) | 2.56 (1.1, 4.25) | 11285 (4804, 18572) | 112.48 (47.85, 184.7) |  | 1.1 (0.88, 1.32) | 1.92 (1.83, 2.01) |
| Eastern Republic of Uruguay | 50 (20, 83) | 1.26 (0.5, 2.09) | | 1337 (553, 2131) | 33.65 (13.95, 53.48) |  | 76 (31, 129) | 1.21 (0.5, 2) | 2675 (1162, 4466) | 49.11 (21.26, 82.13) |  | -0.19 (-0.26, -0.12) | 1.31 (1.2, 1.42) |
| Federal Democratic Republic of Ethiopia | 393 (160, 657) | 2.57 (0.97, 4.4) | | 11513 (4644, 19045) | 61.23 (24.54, 102.25) |  | 568 (212, 982) | 1.64 (0.62, 2.85) | 17517 (6614, 29199) | 42.08 (15.49, 71.26) |  | -1.9 (-2.11, -1.69) | -1.6 (-1.82, -1.39) |
| Federal Democratic Republic of Nepal | 83 (32, 140) | 1.23 (0.47, 2.14) | | 2700 (1074, 4676) | 32.57 (12.65, 56.17) |  | 374 (148, 663) | 2.05 (0.82, 3.77) | 12405 (4715, 21676) | 56.9 (21.81, 99.65) |  | 1.76 (1.51, 2) | 1.9 (1.68, 2.13) |
| Federal Republic of Germany | 2285 (937, 3568) | 1.66 (0.67, 2.59) | | 42851 (18363, 66371) | 31.68 (13.47, 49.4) |  | 2236 (955, 3787) | 0.88 (0.39, 1.47) | 59351 (25462, 98656) | 28.71 (12.23, 47.77) |  | -2.45 (-2.68, -2.22) | -0.81 (-1.14, -0.48) |
| Federal Republic of Nigeria | 895 (366, 1432) | 2.55 (1.03, 4.15) | | 23801 (9873, 37814) | 58.09 (24.17, 90.97) |  | 1917 (753, 3108) | 2.85 (1.12, 4.64) | 58639 (23389, 94739) | 69.6 (27.76, 114.41) |  | 0.33 (0.3, 0.36) | 0.52 (0.48, 0.55) |
| Federal Republic of Somalia | 32 (12, 58) | 1.99 (0.79, 3.44) | | 923 (335, 1656) | 44.4 (17.26, 77.27) |  | 90 (32, 159) | 2.29 (0.8, 4.08) | 2797 (969, 4978) | 52.7 (19.13, 91.05) |  | 0.56 (0.46, 0.65) | 0.6 (0.53, 0.68) |
| Federated States of Micronesia | 7 (3, 10) | 14.38 (5.99, 22.98) | | 213 (90, 343) | 425.34 (180.43, 678.28) |  | 13 (6, 21) | 19.37 (8.12, 30.34) | 519 (229, 811) | 635.85 (283.56, 980.43) |  | 0.91 (0.62, 1.19) | 1.29 (1.04, 1.54) |
| Federative Republic of Brazil | 2929 (1317, 4446) | 3.96 (1.76, 6) | | 91459 (41149, 144606) | 107.84 (48.01, 166.82) |  | 8137 (3344, 12513) | 3.39 (1.4, 5.21) | 270620 (111518, 422465) | 108.46 (44.82, 169.78) |  | -0.39 (-0.55, -0.23) | 0.05 (-0.02, 0.11) |
| French Republic | 1076 (463, 1682) | 1.18 (0.51, 1.85) | | 22700 (9597, 36284) | 26.16 (11.02, 41.94) |  | 1714 (742, 2710) | 0.87 (0.38, 1.37) | 41692 (19178, 66512) | 28.44 (13.08, 45.57) |  | -1 (-1.53, -0.46) | 0.25 (-0.08, 0.59) |
| Gabonese Republic | 9 (4, 17) | 2.08 (0.75, 3.67) | | 224 (85, 383) | 43.28 (16.53, 75.2) |  | 18 (6, 33) | 2.47 (0.9, 4.73) | 498 (190, 881) | 55.07 (20.69, 98.39) |  | 0.4 (0.23, 0.58) | 0.64 (0.49, 0.79) |
| Georgia | 41 (17, 66) | 0.68 (0.29, 1.08) | | 1427 (605, 2313) | 23.09 (9.94, 37.16) |  | 83 (34, 134) | 1.3 (0.52, 2.1) | 2821 (1116, 4744) | 46.47 (18.21, 79.01) |  | 3.46 (2.71, 4.22) | 3.04 (2.62, 3.46) |
| Grand Duchy of Luxembourg | 7 (3, 11) | 1.29 (0.52, 2.02) | | 169 (70, 267) | 30.69 (12.65, 47.69) |  | 8 (4, 14) | 0.68 (0.28, 1.1) | 321 (135, 523) | 29.84 (12.6, 48.38) |  | -2.01 (-2.17, -1.85) | -0.2 (-0.32, -0.07) |
| Greenland | 0 (0, 0) | 1.21 (0.53, 2.05) | | 7 (3, 11) | 24.29 (9.66, 39.41) |  | 0 (0, 1) | 0.64 (0.26, 1.06) | 16 (6, 27) | 24.64 (9.85, 41.07) |  | -2.29 (-2.47, -2.11) | -0.21 (-0.5, 0.07) |
| Grenada | 7 (3, 11) | 8.05 (3.28, 13.46) | | 141 (57, 234) | 190.54 (77.58, 314.8) |  | 7 (3, 12) | 7.74 (3.19, 12.33) | 223 (93, 365) | 204.97 (86.5, 329.18) |  | 0.28 (-0.06, 0.62) | 0.32 (0.13, 0.52) |
| Guam | 2 (1, 3) | 3.29 (1.38, 5.5) | | 54 (23, 88) | 78.25 (32.82, 128.48) |  | 2 (1, 4) | 1.04 (0.45, 1.73) | 116 (49, 193) | 55.33 (23.17, 91.71) |  | -3.33 (-3.61, -3.04) | -1.13 (-1.39, -0.87) |
| Hashemite Kingdom of Jordan | 63 (26, 102) | 6.33 (2.69, 10.04) | | 2032 (849, 3361) | 167.59 (69.78, 271.23) |  | 239 (97, 385) | 4.49 (1.82, 7.21) | 12117 (4883, 19908) | 167.71 (65.22, 274.33) |  | -1.64 (-2.1, -1.18) | -0.32 (-0.61, -0.04) |
| Hellenic Republic | 124 (53, 196) | 0.85 (0.36, 1.33) | | 3886 (1722, 6392) | 25.38 (11.34, 41.37) |  | 188 (79, 304) | 0.59 (0.25, 0.93) | 7268 (2950, 12071) | 29.7 (12.48, 48.7) |  | -1.16 (-1.62, -0.7) | 0.3 (0.08, 0.52) |
| Hungary | 159 (66, 255) | 1.08 (0.45, 1.72) | | 5613 (2375, 9043) | 37.31 (15.65, 60.33) |  | 271 (112, 421) | 1.23 (0.5, 1.91) | 10169 (4107, 16463) | 49.91 (20.04, 81.76) |  | 0.69 (0.31, 1.06) | 1.06 (0.83, 1.29) |
| Independent State of Papua New Guinea | 96 (36, 166) | 6.04 (2.35, 10.33) | | 3346 (1298, 5773) | 170.48 (66.88, 288.65) |  | 275 (105, 483) | 6.23 (2.33, 10.66) | 11770 (4610, 19421) | 205.2 (79.82, 335.52) |  | 0.05 (0.01, 0.1) | 0.55 (0.52, 0.59) |
| Independent State of Samoa | 6 (3, 10) | 8.17 (3.68, 13.09) | | 219 (101, 346) | 248.94 (114.28, 390.97) |  | 15 (7, 25) | 11.23 (4.78, 18.46) | 600 (270, 972) | 394.51 (178.11, 643.62) |  | 1.01 (0.92, 1.1) | 1.48 (1.41, 1.56) |
| Ireland | 58 (26, 89) | 1.44 (0.65, 2.21) | | 1481 (660, 2298) | 35.69 (15.91, 55.29) |  | 59 (26, 95) | 0.68 (0.3, 1.09) | 2515 (1079, 4103) | 31.7 (13.59, 51.66) |  | -2.22 (-2.45, -1.99) | -0.38 (-0.49, -0.27) |
| Islamic Republic of Afghanistan | 184 (68, 299) | 2.91 (1.07, 4.55) | | 7311 (3008, 11736) | 104.34 (43.06, 164.47) |  | 400 (164, 658) | 5.06 (2.08, 8.23) | 22614 (9583, 37086) | 221.5 (99.11, 355.31) |  | 1.99 (1.91, 2.08) | 2.6 (2.54, 2.65) |
| Islamic Republic of Iran | 282 (115, 460) | 1.56 (0.64, 2.55) | | 12013 (4952, 19561) | 49.74 (20.97, 79.45) |  | 1573 (690, 2505) | 2.35 (1.04, 3.74) | 75814 (32428, 120977) | 97.96 (41.75, 155.31) |  | 1.82 (1.6, 2.04) | 2.58 (2.43, 2.72) |
| Islamic Republic of Mauritania | 28 (10, 47) | 3.28 (1.24, 5.41) | | 823 (325, 1332) | 84.48 (33.01, 135.81) |  | 76 (31, 131) | 4.15 (1.69, 7.24) | 2379 (1032, 4045) | 111.31 (48.03, 189.08) |  | 0.54 (0.45, 0.63) | 0.72 (0.65, 0.78) |
| Islamic Republic of Pakistan | 1062 (445, 1763) | 2.27 (0.94, 3.75) | | 31491 (13372, 51877) | 58.81 (24.87, 96.41) |  | 3626 (1516, 5891) | 3.95 (1.63, 6.47) | 128080 (54171, 208118) | 111.11 (48.13, 179.85) |  | 1.55 (1.29, 1.81) | 1.95 (1.72, 2.19) |
| Jamaica | 162 (70, 250) | 8.75 (3.79, 13.44) | | 4029 (1746, 6353) | 223.75 (96.65, 351.51) |  | 288 (125, 470) | 8.9 (3.89, 14.56) | 7487 (3404, 11631) | 241.47 (109.89, 376.65) |  | -0.21 (-0.45, 0.04) | -0.05 (-0.23, 0.13) |
| Japan | 1257 (524, 1979) | 0.77 (0.33, 1.21) | | 66895 (29082, 104582) | 39.52 (17.12, 62.18) |  | 1135 (472, 1822) | 0.23 (0.1, 0.36) | 143365 (58708, 238895) | 48.38 (19.96, 81.07) |  | -3.78 (-4.41, -3.14) | 0.58 (0.45, 0.71) |
| Kingdom of Bahrain | 14 (6, 22) | 13.74 (5.51, 21.95) | | 442 (178, 707) | 300.2 (123.93, 471.14) |  | 78 (34, 124) | 17.33 (7.29, 27.94) | 3097 (1318, 4970) | 402.78 (169.67, 635.22) |  | 0.57 (0.12, 1.01) | 0.68 (0.39, 0.97) |
| Kingdom of Belgium | 242 (97, 391) | 1.51 (0.61, 2.41) | | 5880 (2424, 9553) | 37.26 (15.59, 60.13) |  | 185 (86, 295) | 0.61 (0.29, 0.97) | 8505 (3850, 14107) | 36.75 (16, 60.94) |  | -2.99 (-3.23, -2.75) | -0.36 (-0.5, -0.23) |

**Supplementary Table S2 (Continued).**

| **Characteristics** | **1990** | | | |  | **2021** | | | |  | **EAPC (1990-2021)** | |
| --- | --- | --- | --- | --- | --- | --- | --- | --- | --- | --- | --- | --- |
|  | **Deaths cases No.(95%UI)** | **ASMR per 10^5^ No.(95%UI)** | **DALYs**  **No.(95%UI)** | **ASDR per 10^5^ No.(95%UI)** |  | **Deaths cases No.(95%UI)** | **ASMR per 10^5^ No.(95%UI)** | **DALYs**  **No.(95%UI)** | **ASDR per 10^5^ No.(95%UI)** |  | **ASMR**  **No.(95%CI)** | **ASDR**  **No.(95%CI)** |
| Kingdom of Bhutan | 7 (3, 12) | 3.91 (1.69, 6.37) | 261 (116, 419) | 109.51 (48.5, 175.22) |  | 30 (13, 50) | 5.44 (2.37, 9.08) | 962 (435, 1506) | 158.92 (72.05, 250.9) |  | 1.1 (1.07, 1.13) | 1.21 (1.19, 1.24) |
| Kingdom of Cambodia | 30 (11, 53) | 0.91 (0.31, 1.65) | 809 (281, 1397) | 20.98 (7.55, 36.44) |  | 104 (34, 188) | 1.17 (0.39, 2.1) | 3274 (1208, 5885) | 31.4 (11.53, 55.52) |  | 0.7 (0.47, 0.92) | 1.21 (1.01, 1.41) |
| Kingdom of Denmark | 85 (38, 134) | 0.96 (0.42, 1.5) | 1896 (820, 3000) | 22.81 (9.73, 36.04) |  | 154 (68, 254) | 1.1 (0.49, 1.82) | 3761 (1704, 6156) | 30.51 (13.69, 49.79) |  | 0.41 (-0.14, 0.95) | 0.86 (0.55, 1.17) |
| Kingdom of Eswatini | 15 (6, 25) | 6.55 (2.8, 10.92) | 383 (166, 631) | 147.16 (62.92, 241.6) |  | 45 (17, 79) | 10.16 (3.97, 17.71) | 1296 (494, 2191) | 247.51 (98.23, 419.99) |  | 1.86 (1.25, 2.48) | 2.07 (1.48, 2.66) |
| Kingdom of Lesotho | 16 (6, 27) | 2.32 (0.95, 3.94) | 324 (137, 563) | 43.96 (18.35, 74.58) |  | 37 (14, 67) | 4.94 (1.99, 8.8) | 855 (334, 1513) | 97.19 (38.91, 168.14) |  | 3.39 (2.82, 3.96) | 3.39 (2.89, 3.9) |
| Kingdom of Morocco | 183 (74, 292) | 1.44 (0.6, 2.31) | 9545 (3915, 15692) | 67.45 (27.71, 110.46) |  | 916 (375, 1480) | 2.99 (1.22, 4.79) | 58716 (24825, 93791) | 168 (70.59, 267.58) |  | 2.82 (2.65, 2.99) | 3.18 (3.1, 3.26) |
| Kingdom of Norway | 56 (22, 93) | 0.73 (0.29, 1.19) | 1803 (744, 2957) | 26.04 (10.66, 42.08) |  | 72 (29, 114) | 0.59 (0.24, 0.93) | 2787 (1170, 4445) | 27.98 (11.72, 45.53) |  | -0.82 (-1.35, -0.29) | 0.07 (-0.09, 0.23) |
| Kingdom of Saudi Arabia | 143 (61, 238) | 3.07 (1.32, 5.14) | 5406 (2353, 8883) | 96.14 (42.18, 156.92) |  | 555 (224, 904) | 3.99 (1.66, 6.47) | 34825 (14042, 56333) | 161.64 (66.86, 253.62) |  | 0.64 (0.5, 0.79) | 1.59 (1.52, 1.65) |
| Kingdom of Spain | 1219 (504, 1950) | 2.22 (0.93, 3.58) | 32397 (14049, 50943) | 57.98 (25.27, 90.87) |  | 1238 (532, 2017) | 0.89 (0.38, 1.43) | 52663 (22464, 86857) | 52.59 (22.15, 86.26) |  | -3.02 (-3.19, -2.85) | -0.51 (-0.6, -0.43) |
| Kingdom of Sweden | 143 (59, 229) | 0.84 (0.35, 1.34) | 3627 (1580, 5738) | 23.28 (10.06, 36.92) |  | 198 (83, 325) | 0.71 (0.3, 1.17) | 6308 (2724, 10189) | 29.65 (12.59, 47.86) |  | -0.38 (-0.65, -0.12) | 0.93 (0.84, 1.02) |
| Kingdom of Thailand | 545 (206, 926) | 1.92 (0.74, 3.22) | 15932 (6041, 27803) | 49.73 (19.33, 84.3) |  | 2096 (880, 3488) | 1.93 (0.81, 3.18) | 70948 (29927, 114853) | 65.27 (27.51, 105.65) |  | -0.65 (-0.88, -0.41) | 0.39 (0.23, 0.55) |
| Kingdom of the Netherlands | 342 (133, 548) | 1.66 (0.64, 2.67) | 6991 (2892, 11234) | 33.92 (14.01, 55.02) |  | 296 (118, 494) | 0.72 (0.29, 1.2) | 8352 (3472, 13800) | 22.8 (9.58, 37.16) |  | -2.82 (-3.16, -2.48) | -1.53 (-1.75, -1.31) |
| Kingdom of Tonga | 5 (2, 7) | 8.88 (3.54, 14.17) | 149 (62, 237) | 258.64 (106.56, 419.09) |  | 9 (3, 14) | 10.96 (4.49, 17.58) | 280 (120, 441) | 343.51 (146, 536.33) |  | 0.74 (0.62, 0.86) | 0.91 (0.83, 1) |
| Kyrgyz Republic | 8 (3, 14) | 0.31 (0.12, 0.49) | 398 (168, 678) | 13.78 (5.89, 23.17) |  | 20 (8, 33) | 0.48 (0.19, 0.79) | 1171 (470, 2043) | 25.46 (10.59, 43.83) |  | 0.56 (0.09, 1.03) | 1.53 (1.3, 1.77) |
| Lao People's Democratic Republic | 25 (10, 44) | 1.48 (0.55, 2.59) | 754 (284, 1268) | 38.51 (14.71, 64.92) |  | 57 (22, 101) | 1.57 (0.62, 2.89) | 2061 (811, 3593) | 48.97 (19.14, 86.21) |  | -0.05 (-0.14, 0.03) | 0.54 (0.44, 0.65) |
| Lebanese Republic | 75 (31, 130) | 4.16 (1.72, 6.93) | 2559 (1068, 4187) | 121.98 (50.72, 201.11) |  | 222 (92, 367) | 3.39 (1.39, 5.6) | 9205 (3874, 15221) | 151.24 (62.97, 249.64) |  | -0.35 (-0.51, -0.18) | 0.96 (0.86, 1.06) |
| Malaysia | 210 (90, 337) | 2.53 (1.08, 4.05) | 7435 (3198, 11938) | 83 (35.16, 133.53) |  | 514 (207, 829) | 2.02 (0.81, 3.25) | 27976 (11596, 45555) | 99.11 (41.21, 159.11) |  | -0.97 (-1.19, -0.75) | 0.46 (0.32, 0.59) |
| Mongolia | 2 (1, 3) | 0.2 (0.08, 0.35) | 83 (34, 144) | 8.09 (3.37, 14.14) |  | 6 (2, 11) | 0.32 (0.13, 0.55) | 354 (146, 613) | 15.57 (6.3, 26.92) |  | 1.64 (1.51, 1.76) | 2.14 (2.04, 2.24) |
| Montenegro | 6 (2, 9) | 0.98 (0.4, 1.65) | 228 (93, 373) | 37.56 (15.33, 60.99) |  | 15 (6, 25) | 1.7 (0.67, 2.72) | 584 (245, 971) | 59.67 (25.1, 98.93) |  | 1.87 (1.64, 2.09) | 1.52 (1.43, 1.61) |
| New Zealand | 43 (19, 64) | 1.09 (0.48, 1.63) | 1340 (583, 2137) | 33.68 (14.62, 53.87) |  | 85 (37, 133) | 0.92 (0.4, 1.44) | 3705 (1669, 6013) | 43.89 (19.81, 71.2) |  | -1.21 (-1.67, -0.74) | 0.78 (0.64, 0.92) |
| North Macedonia | 28 (12, 48) | 1.73 (0.71, 2.88) | 918 (371, 1510) | 52.05 (21.1, 84.53) |  | 83 (35, 140) | 3.11 (1.31, 5.25) | 2764 (1164, 4542) | 86.52 (36.59, 141.72) |  | 2 (1.49, 2.51) | 1.71 (1.4, 2.02) |
| Northern Mariana Islands | 1 (0, 1) | 5.51 (2.25, 9.46) | 29 (12, 49) | 149.68 (61.32, 251.56) |  | 2 (1, 4) | 5.08 (2.18, 8.16) | 93 (40, 150) | 166.68 (72.43, 264.83) |  | -0.33 (-0.68, 0.02) | 0.26 (0.01, 0.51) |
| Palestine | 47 (20, 77) | 6.65 (2.87, 10.8) | 1262 (526, 2003) | 154.66 (65.12, 245.93) |  | 126 (52, 200) | 7.02 (2.84, 11.12) | 4779 (2108, 7446) | 199.35 (88.25, 311.94) |  | 0.29 (0.05, 0.53) | 0.91 (0.77, 1.05) |
| People's Democratic Republic of Algeria | 131 (52, 220) | 1.59 (0.65, 2.6) | 6793 (2826, 11038) | 58.86 (24.69, 95.35) |  | 682 (285, 1087) | 2.48 (1.06, 3.97) | 43986 (18350, 71547) | 123.21 (51.71, 196.11) |  | 2.02 (1.82, 2.22) | 2.6 (2.52, 2.67) |
| People's Republic of Bangladesh | 673 (279, 1105) | 1.86 (0.76, 3.1) | 17447 (7099, 28857) | 40.92 (16.94, 67.01) |  | 2949 (1172, 5010) | 2.82 (1.07, 4.77) | 84735 (35639, 140413) | 66.51 (28.08, 109.75) |  | 1.17 (0.72, 1.62) | 1.56 (1.35, 1.76) |
| People's Republic of China | 5532 (2275, 8709) | 0.89 (0.37, 1.4) | 234966 (99510, 371960) | 30.12 (12.64, 46.95) |  | 17106 (7495, 28029) | 0.9 (0.4, 1.47) | 757445 (327375, 1219479) | 36.54 (15.85, 58.04) |  | 0.02 (-0.24, 0.28) | 0.42 (0.29, 0.54) |
| Plurinational State of Bolivia | 77 (29, 126) | 2.86 (1.08, 4.71) | 2062 (805, 3390) | 68.45 (26.46, 112.48) |  | 259 (103, 449) | 3.33 (1.32, 5.64) | 7798 (3118, 13276) | 89.65 (35.49, 151.67) |  | 0.54 (0.51, 0.58) | 0.88 (0.85, 0.91) |
| Portuguese Republic | 359 (151, 558) | 2.67 (1.12, 4.12) | 8898 (3711, 13969) | 63.18 (26.46, 99.5) |  | 546 (223, 873) | 1.68 (0.68, 2.67) | 16576 (7057, 26822) | 65.39 (27.55, 105.81) |  | -1.98 (-2.36, -1.6) | -0.25 (-0.45, -0.05) |
| Principality of Andorra | 1 (0, 1) | 1.33 (0.5, 2.3) | 17 (7, 29) | 31.22 (12.98, 53.22) |  | 2 (1, 3) | 0.94 (0.42, 1.61) | 56 (25, 94) | 35.96 (15.6, 60.87) |  | -0.85 (-1.06, -0.63) | 0.56 (0.41, 0.7) |
| Principality of Monaco | 0 (0, 1) | 0.4 (0.16, 0.68) | 11 (5, 18) | 14.91 (6.28, 25.24) |  | 1 (0, 1) | 0.41 (0.17, 0.69) | 25 (10, 41) | 26.22 (10.65, 44.18) |  | 0.09 (0.07, 0.12) | 1.82 (1.76, 1.88) |
| Puerto Rico | 194 (80, 299) | 5.71 (2.42, 8.8) | 5041 (2075, 8019) | 140.74 (58.51, 221.56) |  | 309 (127, 519) | 3.58 (1.49, 5.95) | 9694 (4034, 16174) | 137.47 (57.64, 230.08) |  | -1.88 (-2.19, -1.56) | -0.41 (-0.65, -0.17) |
| Republic of Albania | 7 (3, 11) | 0.4 (0.17, 0.66) | 346 (144, 565) | 18.33 (7.71, 29.28) |  | 18 (8, 31) | 0.43 (0.18, 0.73) | 1098 (479, 1873) | 24.76 (10.64, 42.38) |  | 0.4 (0.13, 0.68) | 1.09 (1, 1.19) |
| Republic of Angola | 77 (30, 133) | 2.88 (1.09, 4.89) | 2387 (973, 4078) | 69.49 (28.04, 116.16) |  | 246 (92, 439) | 3.1 (1.21, 5.44) | 8824 (3316, 14921) | 82.29 (31.41, 139.72) |  | 0.07 (-0.02, 0.16) | 0.44 (0.38, 0.51) |
| Republic of Armenia | 27 (11, 44) | 1.13 (0.45, 1.84) | 895 (372, 1473) | 34.69 (14.25, 57.04) |  | 47 (19, 76) | 1.08 (0.43, 1.75) | 1676 (693, 2787) | 38.5 (16.27, 63.68) |  | -0.43 (-1.51, 0.67) | -0.02 (-0.78, 0.74) |
| Republic of Austria | 217 (93, 340) | 1.69 (0.72, 2.65) | 4266 (1797, 6686) | 33.85 (14.16, 53.03) |  | 240 (100, 399) | 1.04 (0.44, 1.73) | 5794 (2498, 9354) | 29.85 (12.86, 47.77) |  | -0.83 (-1.34, -0.31) | -0.06 (-0.38, 0.26) |
| Republic of Azerbaijan | 29 (11, 49) | 0.67 (0.25, 1.13) | 1050 (382, 1788) | 22 (8.33, 37.04) |  | 97 (38, 172) | 1.15 (0.47, 2) | 4171 (1534, 7413) | 42.97 (16.34, 73.41) |  | 1.73 (1.34, 2.13) | 2.02 (1.72, 2.32) |
| Republic of Belarus | 35 (15, 58) | 0.28 (0.12, 0.45) | 1642 (696, 2806) | 12.68 (5.39, 21.5) |  | 54 (23, 90) | 0.33 (0.14, 0.54) | 2898 (1206, 4836) | 17.95 (7.44, 30.13) |  | -1.24 (-2.29, -0.18) | 0.44 (0.14, 0.74) |
| Republic of Benin | 16 (6, 27) | 0.95 (0.36, 1.71) | 405 (161, 710) | 22.43 (8.84, 39.32) |  | 52 (20, 94) | 1.42 (0.52, 2.57) | 1634 (640, 2945) | 36.59 (14.22, 66.76) |  | 1.31 (1.14, 1.47) | 1.51 (1.44, 1.59) |
| Republic of Botswana | 31 (13, 52) | 6.94 (2.93, 11.48) | 818 (346, 1391) | 157.72 (68.6, 261.77) |  | 87 (36, 139) | 7.65 (3.14, 12.14) | 2581 (1069, 4030) | 190.81 (79.4, 297.73) |  | 0.69 (0.38, 1.01) | 0.92 (0.66, 1.18) |
| Republic of Bulgaria | 126 (51, 205) | 1.14 (0.47, 1.8) | 4334 (1751, 7000) | 35.19 (14.75, 55.65) |  | 185 (76, 300) | 1.21 (0.49, 1.97) | 7030 (2823, 11674) | 48.11 (19.55, 80.27) |  | -0.15 (-0.47, 0.17) | 0.79 (0.61, 0.97) |
| Republic of Burundi | 40 (16, 71) | 2.1 (0.78, 3.77) | 990 (391, 1757) | 46.04 (18.24, 80.84) |  | 62 (22, 110) | 1.85 (0.69, 3.43) | 1758 (641, 3218) | 41.14 (15.16, 72.69) |  | -0.9 (-1.07, -0.73) | -0.85 (-1.02, -0.68) |
| Republic of Cabo Verde | 1 (0, 2) | 0.51 (0.2, 0.87) | 44 (19, 73) | 18.86 (8.1, 31.25) |  | 8 (3, 15) | 1.98 (0.74, 3.63) | 236 (96, 408) | 55.05 (22.17, 95.88) |  | 3.93 (3.38, 4.49) | 3.31 (2.94, 3.67) |
| Republic of Cameroon | 83 (30, 145) | 2.46 (0.89, 4.29) | 2319 (888, 3985) | 56.78 (21.71, 96.95) |  | 316 (120, 556) | 3.45 (1.33, 5.99) | 10054 (3885, 17321) | 85.92 (33, 146.09) |  | 1.02 (0.79, 1.25) | 1.26 (1.06, 1.47) |
| Republic of Chad | 31 (12, 52) | 1.29 (0.48, 2.21) | 896 (346, 1457) | 33.48 (13.01, 54.86) |  | 89 (34, 152) | 2.04 (0.76, 3.49) | 3110 (1177, 5200) | 56.92 (22.36, 94.67) |  | 1.47 (1.19, 1.75) | 1.69 (1.46, 1.93) |
| Republic of Chile | 92 (38, 159) | 1 (0.42, 1.71) | 2964 (1275, 5107) | 30.1 (13.03, 52.18) |  | 199 (71, 345) | 0.75 (0.27, 1.3) | 10121 (4279, 16864) | 38.96 (16.47, 64.94) |  | -0.75 (-1.17, -0.32) | 0.85 (0.65, 1.05) |
| Republic of Colombia | 316 (134, 490) | 1.99 (0.84, 3.13) | 13525 (5625, 21772) | 74.45 (31.12, 120.64) |  | 735 (317, 1177) | 1.32 (0.57, 2.13) | 46229 (19657, 77574) | 83.53 (35.5, 140.45) |  | -2.19 (-2.5, -1.87) | -0.14 (-0.33, 0.06) |
| Republic of Costa Rica | 16 (6, 26) | 0.97 (0.38, 1.58) | 657 (259, 1077) | 37.92 (14.87, 62.12) |  | 75 (31, 126) | 1.36 (0.56, 2.3) | 3202 (1315, 5230) | 58.24 (23.86, 94.65) |  | 0.1 (-0.69, 0.9) | 1.01 (0.74, 1.29) |
| Republic of Croatia | 65 (28, 102) | 1.23 (0.52, 1.96) | 2216 (946, 3642) | 38.88 (16.38, 63.53) |  | 172 (76, 279) | 1.64 (0.73, 2.66) | 5066 (2342, 8357) | 52.91 (24.29, 87.08) |  | 0.07 (-0.47, 0.61) | 0.61 (0.4, 0.81) |
| Republic of Cuba | 214 (87, 334) | 2.16 (0.88, 3.35) | 6714 (2580, 10630) | 65.76 (25.27, 104.2) |  | 199 (83, 323) | 0.95 (0.4, 1.54) | 11330 (4679, 18753) | 58.36 (24.02, 95.71) |  | -2.73 (-3.42, -2.05) | -0.53 (-0.89, -0.17) |
| Republic of Cyprus | 53 (23, 85) | 9.99 (4.13, 15.61) | 1063 (458, 1696) | 160.11 (68.15, 254.37) |  | 63 (26, 101) | 3.65 (1.52, 5.93) | 1571 (664, 2593) | 79.16 (33.3, 129.56) |  | -3.58 (-3.79, -3.37) | -2.62 (-2.74, -2.49) |
| Republic of Côte d'Ivoire | 51 (19, 89) | 1.87 (0.71, 3.23) | 1645 (622, 2785) | 46.26 (17.55, 78.5) |  | 210 (80, 371) | 2.64 (0.99, 4.75) | 7504 (2978, 12777) | 72.24 (28.97, 123.69) |  | 1.12 (0.88, 1.36) | 1.45 (1.28, 1.62) |
| Republic of Djibouti | 1 (0, 2) | 1.52 (0.57, 2.74) | 36 (13, 64) | 32.29 (11.85, 57.14) |  | 8 (3, 15) | 2.05 (0.71, 3.91) | 243 (90, 416) | 45.15 (17.05, 80.69) |  | 0.96 (0.88, 1.04) | 1.07 (0.99, 1.14) |
| Republic of Ecuador | 55 (22, 90) | 1.16 (0.46, 1.95) | 1836 (737, 3017) | 35.49 (14.17, 58.54) |  | 236 (83, 414) | 1.53 (0.54, 2.72) | 9523 (3679, 16324) | 58.51 (22.62, 100.55) |  | 0.84 (0.4, 1.28) | 1.46 (1.18, 1.75) |

**Supplementary Table S2 (Continued).**

| **Characteristics** | **1990** | | | |  | **2021** | | | |  | **EAPC (1990-2021)** | |
| --- | --- | --- | --- | --- | --- | --- | --- | --- | --- | --- | --- | --- |
|  | **Deaths cases No.(95%UI)** | **ASMR per 10^5^ No.(95%UI)** | **DALYs**  **No.(95%UI)** | **ASDR per 10^5^ No.(95%UI)** |  | **Deaths cases No.(95%UI)** | **ASMR per 10^5^ No.(95%UI)** | **DALYs**  **No.(95%UI)** | **ASDR per 10^5^ No.(95%UI)** |  | **ASMR**  **No.(95%CI)** | **ASDR**  **No.(95%CI)** |
| Republic of El Salvador | 38 (15, 60) | 1.36 (0.52, 2.14) | 1208 (467, 1954) | 41.11 (15.91, 66.37) |  | 182 (69, 317) | 2.84 (1.08, 4.87) | 5399 (2134, 9255) | 87.65 (34.49, 150.22) |  | 2.3 (2.1, 2.51) | 2.32 (2.17, 2.46) |
| Republic of Equatorial Guinea | 4 (2, 8) | 3.05 (1.2, 5.31) | 125 (50, 214) | 71.05 (28.56, 119.43) |  | 15 (5, 27) | 4.19 (1.57, 7.54) | 471 (185, 854) | 102.39 (40.07, 180.57) |  | 1.23 (1.04, 1.43) | 1.38 (1.19, 1.57) |
| Republic of Estonia | 6 (2, 9) | 0.27 (0.12, 0.43) | 273 (113, 465) | 13.16 (5.43, 22.59) |  | 26 (11, 42) | 0.81 (0.34, 1.28) | 892 (364, 1446) | 31.32 (12.39, 51.49) |  | 2.64 (1.73, 3.55) | 2.52 (2.23, 2.8) |
| Republic of Fiji | 49 (20, 81) | 15.37 (6.26, 25.64) | 1574 (680, 2628) | 411.26 (176.13, 684.66) |  | 152 (66, 254) | 22.83 (10.12, 38.75) | 4818 (2061, 8001) | 604.01 (260.43, 981.23) |  | 1.1 (0.81, 1.38) | 1.09 (0.86, 1.31) |
| Republic of Finland | 55 (24, 89) | 0.76 (0.33, 1.21) | 1882 (819, 2987) | 25.94 (11.22, 41.65) |  | 65 (29, 105) | 0.41 (0.18, 0.66) | 4023 (1665, 6690) | 32.74 (13.38, 55.29) |  | -2.3 (-2.73, -1.86) | 0.59 (0.34, 0.84) |
| Republic of Ghana | 91 (36, 149) | 2.02 (0.81, 3.45) | 2879 (1144, 4646) | 50.03 (20.58, 81.6) |  | 402 (160, 658) | 3.34 (1.35, 5.6) | 13299 (5252, 21519) | 84.85 (34.43, 136.98) |  | 1.98 (1.81, 2.15) | 1.98 (1.83, 2.14) |
| Republic of Guatemala | 19 (8, 32) | 0.86 (0.34, 1.51) | 639 (244, 1112) | 21.74 (8.74, 38.38) |  | 259 (98, 457) | 2.62 (1, 4.74) | 7662 (2741, 13027) | 71.4 (25.72, 121.21) |  | 2.48 (1.94, 3.02) | 3.15 (2.78, 3.52) |
| Republic of Guinea | 30 (12, 52) | 1.12 (0.45, 1.99) | 782 (312, 1311) | 25.41 (10.1, 42.58) |  | 74 (27, 136) | 1.7 (0.63, 3.14) | 2043 (770, 3535) | 39.71 (14.94, 69.38) |  | 1.47 (1.26, 1.68) | 1.42 (1.27, 1.57) |
| Republic of Guinea-Bissau | 8 (3, 14) | 2.57 (0.94, 4.53) | 242 (94, 438) | 64.95 (25.44, 114.05) |  | 19 (7, 31) | 3.78 (1.43, 6.38) | 660 (245, 1084) | 98.31 (36.27, 162.61) |  | 1.34 (1.18, 1.51) | 1.39 (1.25, 1.53) |
| Republic of Guyana | 23 (10, 36) | 7.01 (2.95, 10.75) | 754 (308, 1219) | 202.35 (83.55, 321.18) |  | 43 (18, 72) | 7.85 (3.27, 12.58) | 1649 (665, 2705) | 257.71 (103.98, 415.05) |  | 0.12 (-0.33, 0.57) | 0.55 (0.2, 0.9) |
| Republic of Haiti | 212 (83, 343) | 8.05 (3.09, 13.32) | 6473 (2525, 10599) | 207.69 (80.49, 338.48) |  | 448 (174, 770) | 7.84 (3.1, 13.17) | 16093 (6266, 27289) | 229.15 (90.22, 385.1) |  | -0.01 (-0.05, 0.04) | 0.39 (0.35, 0.43) |
| Republic of Honduras | 16 (6, 27) | 0.92 (0.35, 1.52) | 742 (280, 1320) | 36.64 (14.04, 65.21) |  | 110 (45, 190) | 2 (0.84, 3.4) | 4788 (1880, 8140) | 75.81 (30.15, 128.33) |  | 2.62 (2.34, 2.89) | 2.44 (2.31, 2.58) |
| Republic of Iceland | 2 (1, 3) | 0.58 (0.24, 0.92) | 53 (23, 85) | 18.29 (7.87, 29.4) |  | 3 (1, 5) | 0.45 (0.18, 0.73) | 163 (65, 266) | 28.59 (11.27, 46.66) |  | -0.76 (-0.92, -0.61) | 1.45 (1.39, 1.5) |
| Republic of India | 7587 (3202, 11966) | 2.29 (0.96, 3.61) | 235443 (101614, 364556) | 57.84 (25, 90.75) |  | 31512 (13431, 48852) | 3.28 (1.38, 5.06) | 898973 (416117, 1424271) | 81.25 (36.84, 128.62) |  | 1.32 (1.07, 1.58) | 1.05 (0.89, 1.22) |
| Republic of Indonesia | 2641 (1115, 4037) | 3.26 (1.4, 5.02) | 87751 (37733, 135518) | 94.77 (41.33, 145.59) |  | 9293 (4192, 14527) | 4.92 (2.24, 7.61) | 337091 (148073, 528450) | 148.93 (65.61, 231.52) |  | 1.34 (1.24, 1.43) | 1.43 (1.39, 1.48) |
| Republic of Iraq | 364 (146, 591) | 4.94 (1.98, 8.06) | 13562 (5577, 21259) | 168.4 (68.05, 265.23) |  | 1185 (497, 1965) | 6.27 (2.66, 10.35) | 64459 (26895, 101205) | 256.77 (108.09, 402.76) |  | 0.4 (0.2, 0.61) | 1.25 (1.17, 1.34) |
| Republic of Italy | 2383 (1007, 3755) | 2.57 (1.09, 4.03) | 54329 (23262, 84024) | 59.23 (25.51, 91.49) |  | 2633 (1159, 4178) | 1.33 (0.58, 2.08) | 69355 (30643, 111666) | 45.61 (19.8, 74.29) |  | -1.9 (-2.02, -1.78) | -0.69 (-0.78, -0.6) |
| Republic of Kazakhstan | 54 (22, 89) | 0.45 (0.18, 0.74) | 2823 (1144, 4764) | 22.43 (9.06, 37.37) |  | 100 (41, 166) | 0.64 (0.28, 1.08) | 7373 (3079, 12639) | 41.01 (17.49, 70.41) |  | 0.05 (-0.44, 0.54) | 1.43 (1.2, 1.66) |
| Republic of Kenya | 103 (43, 169) | 1.54 (0.64, 2.55) | 2870 (1126, 4631) | 36.55 (15.16, 59.66) |  | 379 (150, 642) | 2.18 (0.84, 3.78) | 11329 (4468, 18396) | 51.82 (20.7, 84.97) |  | 1.4 (1.3, 1.51) | 1.36 (1.25, 1.46) |
| Republic of Kiribati | 6 (2, 9) | 16.62 (6.9, 26.89) | 186 (79, 289) | 470.94 (202.56, 741.34) |  | 14 (6, 23) | 22.2 (9.08, 37.1) | 512 (218, 844) | 649.74 (274.52, 1059.85) |  | 0.87 (0.69, 1.04) | 0.99 (0.82, 1.16) |
| Republic of Korea | 477 (204, 755) | 1.98 (0.85, 3.16) | 18612 (8044, 30118) | 62.98 (26.92, 100.17) |  | 1178 (480, 1952) | 1.27 (0.52, 2.12) | 72367 (30804, 123954) | 78.7 (33.45, 133.32) |  | -2.01 (-2.66, -1.35) | 0.29 (0.05, 0.54) |
| Republic of Latvia | 14 (6, 22) | 0.38 (0.16, 0.61) | 616 (253, 1000) | 17.17 (7.23, 27.91) |  | 45 (18, 74) | 0.99 (0.41, 1.63) | 1563 (670, 2511) | 39.94 (16.89, 64.51) |  | 2.94 (2.36, 3.52) | 2.6 (2.34, 2.85) |
| Republic of Liberia | 23 (9, 38) | 2.43 (0.99, 4) | 725 (297, 1164) | 64.44 (26.48, 102.6) |  | 63 (26, 110) | 3.74 (1.56, 6.49) | 2423 (993, 4042) | 109.97 (45.11, 182.11) |  | 1.53 (1.36, 1.71) | 1.83 (1.69, 1.97) |
| Republic of Lithuania | 13 (5, 21) | 0.29 (0.12, 0.46) | 686 (286, 1144) | 15.17 (6.27, 25.1) |  | 58 (24, 94) | 0.88 (0.37, 1.43) | 2174 (914, 3591) | 37.62 (15.78, 62.34) |  | 2.81 (1.8, 3.82) | 2.62 (2.27, 2.97) |
| Republic of Madagascar | 55 (20, 96) | 1.39 (0.55, 2.44) | 1423 (507, 2517) | 30.88 (11.51, 54.7) |  | 109 (44, 196) | 1.49 (0.59, 2.74) | 3442 (1328, 6083) | 34.96 (14.56, 60.56) |  | 0.17 (0.11, 0.22) | 0.35 (0.3, 0.39) |
| Republic of Malawi | 47 (19, 79) | 1.58 (0.64, 2.75) | 1200 (495, 2109) | 34.44 (13.88, 58.47) |  | 100 (40, 181) | 1.74 (0.69, 3.15) | 2666 (1050, 4857) | 39.5 (15.46, 71.06) |  | 0.09 (-0.13, 0.31) | 0.19 (-0.02, 0.4) |
| Republic of Maldives | 5 (2, 7) | 5.91 (2.58, 9.04) | 162 (70, 243) | 174.48 (75.76, 265.69) |  | 9 (4, 14) | 3.18 (1.39, 5.02) | 457 (198, 728) | 137.77 (59.59, 218.62) |  | -2.37 (-2.55, -2.19) | -1.07 (-1.28, -0.85) |
| Republic of Mali | 70 (30, 114) | 2.29 (1, 3.84) | 2441 (1101, 3984) | 65.54 (29.04, 108.56) |  | 205 (83, 353) | 3.07 (1.22, 5.15) | 8590 (3647, 14685) | 101.61 (43.37, 171.56) |  | 1.02 (0.93, 1.12) | 1.5 (1.43, 1.57) |
| Republic of Malta | 14 (6, 21) | 3.4 (1.47, 5.19) | 297 (132, 458) | 70.72 (31.27, 109.29) |  | 21 (9, 34) | 1.86 (0.78, 2.94) | 695 (299, 1100) | 71.94 (31.16, 112.35) |  | -2.01 (-2.36, -1.65) | 0.03 (-0.27, 0.33) |
| Republic of Mauritius | 22 (9, 35) | 3.29 (1.39, 5.23) | 711 (285, 1185) | 98.81 (41.48, 160.61) |  | 132 (55, 217) | 7.43 (3.09, 12.2) | 3842 (1613, 6331) | 205.76 (86.32, 336.4) |  | 4.15 (3.16, 5.15) | 3.46 (2.76, 4.16) |
| Republic of Moldova | 18 (7, 29) | 0.44 (0.18, 0.72) | 862 (359, 1471) | 20.07 (8.34, 34.11) |  | 39 (16, 64) | 0.64 (0.26, 1.05) | 2031 (827, 3461) | 33.85 (13.67, 57.78) |  | 0.28 (-0.65, 1.21) | 1.3 (1.03, 1.58) |
| Republic of Mozambique | 66 (24, 115) | 1.51 (0.58, 2.68) | 1737 (647, 3060) | 32.76 (12.14, 57.27) |  | 155 (57, 281) | 1.99 (0.73, 3.53) | 4599 (1756, 8022) | 46.1 (17.62, 81.82) |  | 1.33 (1.18, 1.49) | 1.51 (1.37, 1.66) |
| Republic of Namibia | 28 (11, 46) | 5.41 (2.2, 8.93) | 732 (311, 1194) | 121.6 (50.76, 199.14) |  | 76 (29, 125) | 6.92 (2.72, 11.36) | 2025 (819, 3302) | 159.4 (64.94, 259.03) |  | 0.56 (0.26, 0.87) | 0.65 (0.37, 0.93) |
| Republic of Nauru | 0 (0, 1) | 9.12 (3.41, 15.31) | 14 (5, 23) | 268.95 (101.45, 453.54) |  | 1 (0, 1) | 12.56 (4.93, 20.91) | 25 (10, 41) | 395.51 (155.09, 646.15) |  | 0.93 (0.75, 1.1) | 1.1 (0.91, 1.28) |
| Republic of Nicaragua | 21 (8, 35) | 1.55 (0.63, 2.64) | 754 (316, 1284) | 50.34 (20.71, 85.72) |  | 87 (33, 155) | 1.97 (0.74, 3.54) | 3715 (1471, 6488) | 76.45 (30.47, 133.66) |  | 0.84 (0.6, 1.07) | 1.31 (1.18, 1.45) |
| Republic of Niue | 0 (0, 0) | 7.15 (2.77, 11.7) | 5 (2, 7) | 207.52 (85.49, 331.89) |  | 0 (0, 0) | 10.65 (4.3, 18.25) | 7 (3, 12) | 332.22 (137.26, 542.8) |  | 1.17 (1.02, 1.33) | 1.38 (1.24, 1.52) |
| Republic of Palau | 1 (0, 1) | 7.04 (2.86, 11.85) | 21 (8, 34) | 209.03 (84.8, 339.32) |  | 2 (1, 3) | 8.99 (3.64, 14.86) | 67 (29, 112) | 284.22 (120.63, 477.99) |  | 0.95 (0.8, 1.1) | 1.05 (0.94, 1.16) |
| Republic of Panama | 20 (8, 31) | 1.43 (0.59, 2.27) | 630 (251, 1016) | 43.21 (17.3, 69.8) |  | 87 (37, 145) | 1.93 (0.83, 3.21) | 3019 (1258, 5078) | 68.18 (28.3, 114.97) |  | 0.81 (0.52, 1.09) | 1.32 (1.16, 1.48) |
| Republic of Paraguay | 34 (12, 59) | 1.73 (0.64, 3) | 959 (357, 1680) | 45.24 (17.02, 79.21) |  | 201 (76, 351) | 3.77 (1.43, 6.54) | 5485 (2269, 9388) | 97.12 (39.73, 164.47) |  | 2.92 (2.62, 3.22) | 2.73 (2.49, 2.97) |
| Republic of Peru | 110 (46, 179) | 1.03 (0.43, 1.68) | 3269 (1353, 5391) | 28.61 (11.93, 46.74) |  | 435 (171, 753) | 1.33 (0.52, 2.29) | 14076 (5821, 23466) | 42.54 (17.59, 70.7) |  | 0.58 (0.31, 0.85) | 1.15 (0.98, 1.32) |
| Republic of Poland | 507 (219, 792) | 1.15 (0.5, 1.78) | 20747 (9164, 34085) | 46.89 (20.75, 77.18) |  | 1070 (452, 1675) | 1.36 (0.58, 2.14) | 43280 (18908, 69689) | 58.99 (25.69, 95.91) |  | 0.79 (0.44, 1.14) | 0.91 (0.7, 1.13) |
| Republic of Rwanda | 47 (18, 82) | 2.23 (0.84, 3.92) | 1222 (473, 2143) | 47.84 (18.37, 82.62) |  | 80 (28, 156) | 1.84 (0.63, 3.68) | 2091 (739, 4101) | 38.52 (13.74, 73.09) |  | -1.44 (-1.75, -1.13) | -1.55 (-1.86, -1.23) |
| Republic of San Marino | 0 (0, 1) | 0.91 (0.37, 1.47) | 8 (4, 13) | 23.39 (9.94, 36.64) |  | 0 (0, 1) | 0.44 (0.18, 0.77) | 21 (9, 36) | 29.41 (11.87, 50.07) |  | -1.54 (-1.86, -1.21) | 0.97 (0.88, 1.07) |
| Republic of Senegal | 61 (24, 99) | 2.31 (0.9, 3.78) | 1974 (787, 3228) | 64.3 (25.68, 103.06) |  | 228 (90, 387) | 3.65 (1.43, 6.26) | 8038 (3561, 13504) | 108.58 (47.58, 180.98) |  | 1.46 (1.32, 1.6) | 1.76 (1.63, 1.89) |
| Republic of Serbia | 156 (65, 248) | 1.87 (0.79, 2.97) | 5214 (2068, 8276) | 52.03 (20.76, 82.23) |  | 348 (141, 571) | 1.94 (0.78, 3.19) | 11611 (4799, 19035) | 67.27 (27.88, 111.43) |  | -0.02 (-0.18, 0.14) | 0.7 (0.59, 0.81) |
| Republic of Seychelles | 1 (0, 1) | 1.41 (0.59, 2.25) | 26 (11, 41) | 45.92 (19.41, 73.09) |  | 2 (1, 4) | 2.35 (0.98, 3.77) | 113 (46, 189) | 104.12 (42.08, 170.56) |  | 2.03 (1.8, 2.26) | 2.84 (2.66, 3.01) |
| Republic of Sierra Leone | 26 (10, 42) | 1.53 (0.58, 2.55) | 747 (310, 1234) | 38.68 (15.86, 64.98) |  | 67 (26, 115) | 2.3 (0.91, 3.93) | 2335 (990, 3837) | 64.91 (27.25, 108.29) |  | 1.49 (1.23, 1.74) | 1.86 (1.66, 2.06) |
| Republic of Singapore | 30 (13, 48) | 1.54 (0.66, 2.52) | 1437 (620, 2327) | 63.07 (27.18, 101.56) |  | 14 (6, 23) | 0.17 (0.07, 0.28) | 4004 (1630, 6796) | 46.06 (18.71, 78.26) |  | -7.03 (-8.09, -5.95) | -1.17 (-1.38, -0.96) |
| Republic of Slovenia | 23 (10, 37) | 0.92 (0.39, 1.5) | 814 (343, 1350) | 32.71 (13.57, 54.36) |  | 39 (15, 61) | 0.72 (0.29, 1.14) | 1517 (630, 2494) | 32.92 (13.82, 53.94) |  | -2.84 (-3.58, -2.11) | -1.02 (-1.35, -0.68) |
| Republic of South Africa | 1098 (482, 1698) | 6 (2.67, 9.26) | 30672 (13413, 47001) | 151.97 (67.1, 232.59) |  | 4220 (1803, 6408) | 10.71 (4.56, 16.37) | 117395 (51482, 178918) | 263.45 (114.42, 400.65) |  | 2.08 (1.63, 2.54) | 1.94 (1.6, 2.28) |
| Republic of South Sudan | 35 (13, 65) | 1.71 (0.63, 3.25) | 843 (297, 1534) | 36.19 (13.9, 67.96) |  | 60 (21, 110) | 2.18 (0.71, 3.92) | 1657 (558, 3087) | 48.26 (16.65, 86.79) |  | 0.73 (0.6, 0.85) | 0.86 (0.72, 1) |
| Republic of Sudan | 184 (80, 293) | 2.17 (0.95, 3.45) | 8837 (4064, 13646) | 91.52 (42.07, 141.81) |  | 567 (246, 947) | 3.24 (1.39, 5.37) | 37326 (17218, 58482) | 171.35 (77.74, 267.77) |  | 1.51 (1.34, 1.68) | 2.16 (2.09, 2.23) |
| Republic of Suriname | 9 (4, 15) | 3.98 (1.73, 6.34) | 337 (144, 536) | 130.53 (55.54, 208.54) |  | 26 (11, 43) | 4.32 (1.85, 7.02) | 1257 (518, 2024) | 194.13 (80.45, 311.58) |  | 0.59 (0.4, 0.78) | 1.51 (1.38, 1.63) |

**Supplementary Table S2 (Continued).**

| **Characteristics** | **1990** | | | |  | **2021** | | | |  | **EAPC (1990-2021)** | |
| --- | --- | --- | --- | --- | --- | --- | --- | --- | --- | --- | --- | --- |
|  | **Deaths cases No.(95%UI)** | **ASMR per 10^5^ No.(95%UI)** | **DALYs**  **No.(95%UI)** | **ASDR per 10^5^ No.(95%UI)** |  | **Deaths cases No.(95%UI)** | **ASMR per 10^5^ No.(95%UI)** | **DALYs**  **No.(95%UI)** | **ASDR per 10^5^ No.(95%UI)** |  | **ASMR**  **No.(95%CI)** | **ASDR**  **No.(95%CI)** |
| Republic of Tajikistan | 17 (7, 27) | 0.66 (0.26, 1.07) | 537 (216, 862) | 20.2 (8.08, 31.84) |  | 39 (14, 66) | 0.84 (0.33, 1.45) | 1779 (696, 3044) | 32.38 (12.97, 55.72) |  | 0.53 (0.17, 0.89) | 1.36 (1.14, 1.59) |
| Republic of the Congo | 26 (11, 43) | 3.12 (1.25, 5.2) | 784 (308, 1297) | 76.02 (30.32, 125.46) |  | 64 (23, 109) | 3.15 (1.07, 5.51) | 2244 (883, 3756) | 83.42 (32.33, 142.05) |  | -0.19 (-0.3, -0.07) | 0.09 (-0.05, 0.23) |
| Republic of the Gambia | 3 (1, 6) | 1.32 (0.5, 2.31) | 98 (39, 165) | 31.26 (12.26, 52.87) |  | 18 (7, 32) | 2.32 (0.89, 4.24) | 538 (209, 912) | 58.74 (22.99, 101.5) |  | 1.78 (1.64, 1.93) | 1.98 (1.86, 2.11) |
| Republic of the Marshall Islands | 3 (1, 4) | 17.64 (7.6, 27.93) | 96 (42, 150) | 551.22 (241.07, 860.01) |  | 9 (4, 15) | 27.56 (10.76, 45.29) | 387 (168, 603) | 943.68 (406.21, 1461.59) |  | 1.33 (1.05, 1.62) | 1.66 (1.41, 1.91) |
| Republic of the Niger | 26 (10, 45) | 1.35 (0.51, 2.36) | 911 (345, 1493) | 36.15 (14.45, 59.68) |  | 108 (41, 190) | 1.84 (0.72, 3.22) | 4238 (1704, 7283) | 55.13 (22.49, 92.76) |  | 1.01 (0.91, 1.12) | 1.37 (1.3, 1.45) |
| Republic of the Philippines | 346 (130, 571) | 1.56 (0.57, 2.63) | 11131 (4386, 18491) | 41.95 (16.52, 67.77) |  | 1508 (638, 2505) | 2.21 (0.91, 3.65) | 47572 (19542, 77638) | 61.39 (25.45, 100.42) |  | 1.33 (1.26, 1.4) | 1.37 (1.3, 1.44) |
| Republic of the Union of Myanmar | 291 (108, 523) | 1.54 (0.59, 2.74) | 8220 (3026, 14681) | 37.96 (14.76, 66.1) |  | 721 (265, 1297) | 1.77 (0.64, 3.25) | 23229 (8557, 39825) | 50.76 (18.52, 87.61) |  | 0.26 (0.13, 0.39) | 0.8 (0.62, 0.98) |
| Republic of Trinidad and Tobago | 120 (52, 181) | 15.71 (6.8, 23.63) | 3600 (1580, 5493) | 435.94 (191.37, 661.57) |  | 228 (101, 374) | 11.86 (5.19, 19.45) | 7699 (3428, 12376) | 390.41 (174.38, 625.18) |  | -1.14 (-1.29, -0.99) | -0.62 (-0.74, -0.5) |
| Republic of Tunisia | 39 (16, 63) | 1.06 (0.44, 1.69) | 1613 (651, 2648) | 35.77 (14.38, 58.16) |  | 201 (82, 334) | 1.72 (0.71, 2.83) | 10224 (3890, 17566) | 79.22 (30.44, 134.37) |  | 1.75 (1.61, 1.89) | 2.62 (2.48, 2.76) |
| Republic of Turkey | 1235 (522, 1916) | 4.46 (1.9, 6.96) | 30926 (12779, 48401) | 99.36 (41.32, 153.64) |  | 2886 (1260, 4666) | 3.45 (1.51, 5.65) | 99529 (42177, 161690) | 108.7 (45.7, 177.1) |  | -0.22 (-0.64, 0.21) | 0.62 (0.31, 0.93) |
| Republic of Uganda | 79 (29, 146) | 1.63 (0.58, 3.16) | 1941 (715, 3623) | 34.13 (12.57, 62.68) |  | 212 (69, 403) | 2.03 (0.64, 3.82) | 5486 (1890, 10128) | 43 (15, 79.59) |  | 0.33 (0.1, 0.56) | 0.33 (0.11, 0.55) |
| Republic of Uzbekistan | 34 (13, 57) | 0.32 (0.12, 0.53) | 1309 (521, 2258) | 11.6 (4.53, 19.78) |  | 213 (89, 372) | 0.9 (0.37, 1.54) | 8939 (3768, 15394) | 33.99 (14.22, 56.75) |  | 3.3 (2.84, 3.76) | 3.5 (3.17, 3.84) |
| Republic of Vanuatu | 1 (0, 2) | 2.24 (0.83, 4.15) | 29 (11, 55) | 52.09 (20.46, 91.53) |  | 4 (1, 7) | 2.99 (1.14, 5.27) | 119 (45, 213) | 74.53 (28.41, 129.16) |  | 0.77 (0.69, 0.84) | 0.91 (0.82, 1.01) |
| Republic of Yemen | 54 (22, 93) | 1.4 (0.55, 2.31) | 2309 (972, 3658) | 48.54 (20.36, 76.94) |  | 205 (80, 346) | 1.8 (0.71, 3.03) | 11686 (4922, 18610) | 81.84 (34.62, 129.51) |  | 0.94 (0.81, 1.08) | 1.81 (1.69, 1.93) |
| Republic of Zambia | 52 (21, 90) | 2.3 (0.97, 4.07) | 1462 (603, 2468) | 55.54 (22.38, 92.36) |  | 119 (47, 215) | 2.31 (0.88, 4.15) | 3982 (1584, 6975) | 61.42 (24.04, 107.89) |  | -0.38 (-0.55, -0.21) | -0.06 (-0.2, 0.09) |
| Republic of Zimbabwe | 79 (31, 133) | 2.63 (1.1, 4.53) | 1988 (780, 3341) | 56.7 (22.68, 95.25) |  | 235 (89, 415) | 4.63 (1.75, 7.95) | 6631 (2561, 11369) | 108.58 (41, 182.54) |  | 2.5 (1.93, 3.07) | 2.72 (2.21, 3.23) |
| Romania | 150 (64, 235) | 0.57 (0.24, 0.9) | 6911 (3015, 11391) | 24.77 (10.8, 40.8) |  | 247 (102, 396) | 0.6 (0.25, 0.96) | 12307 (5068, 21002) | 32.61 (13.21, 55.45) |  | 0.26 (-0.13, 0.64) | 0.99 (0.86, 1.11) |
| Russian Federation | 494 (203, 751) | 0.28 (0.11, 0.43) | 26467 (10780, 42143) | 14.84 (6.1, 23.6) |  | 3641 (1471, 5757) | 1.47 (0.59, 2.34) | 104013 (42716, 164495) | 42.25 (17.23, 66.5) |  | 4.65 (2.9, 6.44) | 3.03 (2.42, 3.65) |
| Saint Kitts and Nevis | 3 (1, 5) | 7.49 (3.27, 11.93) | 70 (30, 114) | 183.78 (80.09, 299.25) |  | 3 (1, 5) | 5.3 (2.14, 8.56) | 106 (44, 176) | 160.55 (66.97, 265.21) |  | -0.6 (-0.93, -0.27) | -0.13 (-0.33, 0.08) |
| Saint Lucia | 8 (3, 12) | 9.99 (4.14, 16.17) | 198 (81, 315) | 236.1 (96.42, 376.07) |  | 13 (6, 22) | 5.77 (2.41, 9.48) | 425 (178, 696) | 177.72 (75.47, 290.5) |  | -3.02 (-3.4, -2.64) | -1.67 (-1.89, -1.44) |
| Saint Vincent and the Grenadines | 7 (3, 11) | 10.39 (4.48, 16.8) | 166 (72, 270) | 238.09 (103.06, 385.27) |  | 9 (4, 14) | 6.77 (2.73, 11.12) | 281 (119, 456) | 199.15 (84.7, 321.3) |  | -1.72 (-2.03, -1.4) | -0.92 (-1.12, -0.72) |
| Slovak Republic | 49 (20, 80) | 0.81 (0.34, 1.34) | 1663 (703, 2710) | 27.44 (11.5, 45.11) |  | 65 (27, 111) | 0.67 (0.28, 1.13) | 2990 (1181, 5042) | 30.85 (12.1, 52.37) |  | -0.56 (-0.64, -0.48) | 0.4 (0.36, 0.44) |
| Socialist Republic of Viet Nam | 601 (232, 1048) | 1.69 (0.66, 2.93) | 15304 (5891, 26742) | 39.94 (15.26, 70.87) |  | 1806 (691, 3280) | 2.18 (0.83, 3.92) | 54981 (21203, 94881) | 59.09 (23.41, 101.14) |  | 0.96 (0.9, 1.03) | 1.46 (1.35, 1.57) |
| Solomon Islands | 10 (4, 17) | 7.92 (3.28, 13.74) | 320 (124, 555) | 216.24 (86.93, 372.73) |  | 34 (13, 57) | 10.85 (4.33, 17.96) | 1240 (500, 2032) | 321.79 (131.77, 524.87) |  | 1.07 (0.99, 1.16) | 1.34 (1.25, 1.43) |
| State of Eritrea | 7 (3, 13) | 1.02 (0.35, 1.97) | 219 (82, 397) | 22.95 (8.47, 42.76) |  | 22 (8, 41) | 1.26 (0.46, 2.48) | 686 (255, 1228) | 28.72 (10.94, 51.31) |  | 0.77 (0.71, 0.83) | 0.83 (0.79, 0.87) |
| State of Israel | 100 (41, 157) | 2.17 (0.9, 3.42) | 2580 (1056, 4104) | 53.13 (21.63, 84.51) |  | 282 (113, 462) | 1.99 (0.81, 3.23) | 7133 (2834, 11307) | 55.74 (22.22, 87.58) |  | -0.89 (-1.76, -0.02) | -0.3 (-0.92, 0.33) |
| State of Kuwait | 15 (7, 23) | 3.4 (1.48, 5.23) | 987 (439, 1569) | 146.45 (65.34, 230.96) |  | 82 (35, 130) | 3.76 (1.6, 6.03) | 8379 (3693, 13697) | 235.71 (104.49, 381.93) |  | -0.01 (-0.56, 0.53) | 1.53 (1.3, 1.77) |
| State of Libya | 27 (11, 44) | 1.63 (0.69, 2.66) | 1186 (515, 1898) | 63.43 (27.1, 101.83) |  | 133 (55, 222) | 3.02 (1.25, 4.97) | 7788 (3370, 12498) | 144.31 (63.61, 232.79) |  | 2.93 (2.61, 3.25) | 3.18 (2.98, 3.38) |
| State of Qatar | 6 (3, 10) | 11.29 (4.55, 18.03) | 240 (99, 377) | 263.98 (110.7, 408.6) |  | 45 (18, 76) | 10.76 (4.42, 18.05) | 3044 (1263, 5172) | 320.12 (129.87, 516.65) |  | -0.24 (-1.12, 0.65) | 0.56 (-0.04, 1.16) |
| Sultanate of Oman | 26 (11, 44) | 4.76 (2.08, 8.05) | 820 (342, 1345) | 127.87 (53.68, 210.74) |  | 78 (31, 125) | 5.93 (2.41, 9.45) | 3372 (1403, 5215) | 173.38 (72.71, 274.27) |  | 1.25 (0.98, 1.53) | 1.22 (1.08, 1.35) |
| Swiss Confederation | 187 (76, 289) | 1.61 (0.65, 2.47) | 4483 (1846, 6990) | 41.18 (17.19, 64.51) |  | 142 (57, 234) | 0.59 (0.23, 0.97) | 7345 (2810, 12232) | 40.85 (15.53, 68.58) |  | -3.56 (-3.81, -3.32) | -0.3 (-0.45, -0.16) |
| Syrian Arab Republic | 96 (38, 155) | 2.24 (0.91, 3.57) | 3739 (1562, 5938) | 73.46 (31.48, 115.25) |  | 259 (109, 440) | 2.53 (1.1, 4.23) | 14969 (6289, 24581) | 114.29 (48.52, 186.71) |  | -0.04 (-0.26, 0.18) | 1.16 (1.03, 1.3) |
| Taiwan (Province of China) | 493 (208, 780) | 3.57 (1.51, 5.63) | 15514 (6474, 24564) | 97.9 (41.38, 153.52) |  | 1089 (452, 1774) | 2.48 (1.02, 4.05) | 38384 (16754, 62288) | 91.19 (39.67, 148.16) |  | -2.2 (-2.73, -1.67) | -1.09 (-1.46, -0.71) |
| Togolese Republic | 9 (3, 16) | 1.05 (0.4, 1.88) | 250 (95, 437) | 23.6 (9.17, 41.35) |  | 41 (15, 70) | 1.66 (0.62, 2.96) | 1217 (484, 2079) | 38.25 (15.17, 65.1) |  | 1.47 (1.31, 1.63) | 1.54 (1.43, 1.65) |
| Tokelau | 0 (0, 0) | 6.55 (2.81, 11.5) | 3 (1, 5) | 205.63 (83.65, 348.87) |  | 0 (0, 0) | 7.23 (2.84, 12.35) | 4 (2, 6) | 263.87 (107.5, 427.48) |  | 0.24 (0.1, 0.37) | 0.7 (0.58, 0.83) |
| Turkmenistan | 10 (4, 15) | 0.57 (0.23, 0.92) | 321 (137, 523) | 17.61 (7.44, 28.56) |  | 44 (17, 75) | 1.24 (0.48, 2.07) | 1610 (643, 2700) | 40.82 (16.3, 67.24) |  | 1.82 (1.41, 2.23) | 2.25 (1.97, 2.53) |
| Tuvalu | 0 (0, 1) | 7.67 (3.02, 12.44) | 15 (6, 24) | 215.8 (84.6, 350.74) |  | 1 (0, 1) | 8.78 (3.41, 14.27) | 29 (11, 45) | 268.27 (105.72, 424.14) |  | 0.43 (0.33, 0.54) | 0.67 (0.58, 0.77) |
| Ukraine | 120 (48, 195) | 0.16 (0.07, 0.27) | 7231 (3073, 12018) | 10.01 (4.31, 16.63) |  | 128 (48, 219) | 0.16 (0.06, 0.27) | 12202 (4648, 20825) | 15.5 (5.94, 26.24) |  | -1.54 (-2.12, -0.96) | 0.97 (0.79, 1.14) |
| Union of the Comoros | 2 (1, 4) | 1.57 (0.57, 2.73) | 66 (26, 109) | 36.73 (14.34, 61.73) |  | 7 (2, 12) | 1.69 (0.62, 3.1) | 204 (77, 358) | 44.13 (16.72, 76.95) |  | 0.12 (0.04, 0.2) | 0.53 (0.41, 0.64) |
| United Arab Emirates | 15 (6, 26) | 5.01 (2.01, 8.69) | 567 (229, 993) | 142.54 (58.09, 244.13) |  | 87 (35, 142) | 5.49 (2.25, 8.93) | 7135 (2820, 11753) | 167.84 (68.95, 267.2) |  | 2.34 (1.61, 3.08) | 1.58 (1.14, 2.01) |
| United Kingdom of Great Britain and Northern Ireland | 1475 (636, 2259) | 1.53 (0.66, 2.33) | 43907 (19502, 67410) | 48.91 (21.73, 74.77) |  | 1135 (471, 1678) | 0.73 (0.31, 1.08) | 81942 (36035, 132236) | 70.13 (30.48, 115.29) |  | -2.36 (-2.67, -2.05) | 1.01 (0.77, 1.24) |
| United Mexican States | 1877 (740, 2890) | 5.17 (2.03, 8.07) | 54317 (22097, 85501) | 132.62 (53.79, 206.56) |  | 5476 (2292, 8653) | 4.66 (1.96, 7.47) | 167331 (69418, 266953) | 132.73 (55.68, 211.56) |  | -0.46 (-0.88, -0.03) | -0.21 (-0.62, 0.19) |
| United Republic of Tanzania | 51 (19, 89) | 0.63 (0.23, 1.13) | 1259 (488, 2186) | 13.12 (4.97, 22.91) |  | 128 (46, 239) | 0.66 (0.23, 1.25) | 3350 (1342, 6227) | 14.78 (5.57, 26.74) |  | -0.14 (-0.26, -0.02) | 0.07 (-0.04, 0.18) |
| United States of America | 4265 (1814, 6821) | 1.26 (0.53, 2.02) | 121255 (51459, 194278) | 37.23 (15.67, 59.73) |  | 6603 (2698, 10503) | 1.05 (0.43, 1.66) | 359724 (148407, 582604) | 61.32 (25.55, 99.54) |  | -1.21 (-1.61, -0.81) | 1.4 (1.27, 1.54) |
| United States Virgin Islands | 3 (1, 4) | 4.35 (1.88, 6.85) | 88 (36, 137) | 114.99 (48.36, 179.34) |  | 5 (2, 8) | 2.56 (1.07, 4.19) | 209 (86, 345) | 113.42 (46.33, 185.31) |  | -1.72 (-1.94, -1.49) | -0.12 (-0.23, -0.02) |

No., number; ASMR, age-standardized mortality rate; *UI*, uncertainty interval;

DALYs, disability-adjusted life years; ASDR, age-standardized DALY rate; EAPC, estimated annual percentage change; *CI*, confidence interval.
